# Structure–Activity Relationship Studies in a Series of 2-Aryloxy-*N*-(pyrimidin-5-yl)acetamide Inhibitors of SLACK Potassium Channels

**DOI:** 10.3390/molecules29235494

**Published:** 2024-11-21

**Authors:** Nigam M. Mishra, Brittany D. Spitznagel, Yu Du, Yasmeen K. Mohamed, Ying Qin, C. David Weaver, Kyle A. Emmitte

**Affiliations:** 1 Department of Pharmaceutical Sciences, UNT System College of Pharmacy, University of North Texas Health Science Center, Fort Worth, TX 76107, USA; 2 Department of Pharmacology, Vanderbilt University, Nashville, TN 37232, USA; 3 Vanderbilt Institute of Chemical Biology, Vanderbilt University, Nashville, TN 37232, USA; 4 College of Biomedical and Translational Sciences, University of North Texas Health Science Center, Fort Worth, TX 76107, USA

**Keywords:** SLACK, *KCNT1*, K_Na_1.1, Slo2.2, CNS, EIMFS, MMPSI, pyrimidine, epilepsy

## Abstract

Epilepsy of infancy with migrating focal seizures (EIMFS) is a rare, serious, and pharmacoresistant epileptic disorder often linked to gain-of-function mutations in the *KCNT1* gene. *KCNT1* encodes the sodium-activated potassium channel known as SLACK, making small molecule inhibitors of SLACK channels a compelling approach to the treatment of EIMFS and other epilepsies associated with *KCNT1* mutations. In this manuscript, we describe a hit optimization effort executed within a series of 2-aryloxy-*N*-(pyrimidin-5-yl)acetamides that were identified via a high-throughput screen. We systematically prepared analogs in four distinct regions of the scaffold and evaluated their functional activity in a whole-cell, automated patch clamp (APC) assay to establish structure-activity relationships for wild-type (WT) SLACK inhibition. Two selected analogs were also profiled for selectivity versus other members of the Slo family of potassium channels, of which SLACK is a member, and versus a panel of structurally diverse ion channels. The same two analogs were evaluated for activity versus the WT mouse channel as well as two clinically relevant mutant human channels.

## 1. Introduction

While rare, epilepsy in infancy with migrating focal seizures (EIMFS), previously known as malignant migrating partial seizures of infancy (MMPSI), remains a devastating disease with extremely limited treatment options [1]. Within six months of birth, most EIMFS patients begin experiencing persistent seizures originating in and migrating across different regions of the brain [2,3,4]. Characteristics of disease progression include a decrease in both motor and cognitive function and development. EIMFS patients also commonly experience other symptoms such as muscle weakness and changes in vision. Prior research has linked gain-of-function (GOF) mutations in the *KCNT1* gene to approximately half of EIMFS cases. The gene product of *KCNT1* is the sodium (Na^+^)-activated potassium (K^+^) channel known as SLACK (sequence like a calcium-activated K^+^ channel). These channels are widely expressed throughout the mammalian central nervous system (CNS) and play an important role in regulating electrical conduction [2,5,6]. Specifically, SLACK manages processes related to neural excitability, such as the afterhyperpolarization (AHP) that occurs after recurrent neural discharges. Proper SLACK function is consequently essential for modulation of action potential frequencies [2,3,4,5,6,7,8].

In addition to SLACK (K_Na_1.1, Slo2.2), there are three additional members of the Slo family of K^+^ channels: Slo1 (Maxi-K, BK, or K_Ca_1.1), Slo2.1 (SLICK), and Slo3 [5,6,9]. Beyond their prevalence in the CNS, SLACK channels are found in other tissues as well, including the gonads, myocytes, and the pituitary glands [2,5,6]. SLACK channels exist as tetramers with each subunit having six hydrophobic transmembrane segments (S1 to S6). Between S5 and S6 is a pore-forming domain. The cytoplasmic *C*-terminus contains a large region that is characterized by two tandem regulators of potassium conductance (RCK) domains. These RCK domains are responsible for providing the channel’s Na^+^ sensitivity. Also present in the C-terminus is a nicotinamide adenine dinucleotide (NAD^+^)-binding domain [3,7,9,10,11,12,13,14]. In addition to intracellular Na^+^ levels, other modulators of SLACK activity have been noted, including membrane potential and phosphorylation by protein kinase A (PKA). Likewise, changing intracellular levels of certain small molecules, including NAD^+^, ATP, estradiol, phosphatidylinositol 4,5-bisphosphate (PIP_2_) and others, can also affect the activity of SLACK channels [12,15,16,17].

To date, over thirty distinct GOF mutations in *KCNT1* have been discovered, and all of them have been linked to EIMFS [2,18,19,20,21]. The majority of these mutations are found in the *C*-terminus or transmembrane pore domain of the channel [2,8,9,13,22,23]. It is of relevance that it has been demonstrated that the cytoplasmic domain of SLACK channels interacts with proteins that are associated with development, including the fragile X mental retardation protein (FMRP), the cytoplasmic FMR1-interacting protein 1 (Cyfip1), and the actin regulator 1 (Phactr1) [24,25]. The impact of GOF mutations in *KCNT1* extends beyond EIMFS to other epileptic disorders, such as autosomal dominant nocturnal frontal lobe epilepsy, West syndrome, and Ohtahara syndrome [23,26,27,28,29]. Researchers have posited multiple explanations for precisely how GOF mutations in *KCNT1* contribute to neuronal excitability. One possible explanation is that these mutations decrease the activity of important inhibitory neurons. On the other hand, GOF mutations in *KCNT1* also cause increased amplitudes of AHP, leading to increased rates of neuronal firing. Still, others point toward potential alterations to appropriate cell signaling as a potential explanation. Of course, one cannot rule out the possibility of multiple mechanisms that contribute to the observed epileptic phenotype [13,18,21,30].

**Figure 1:**
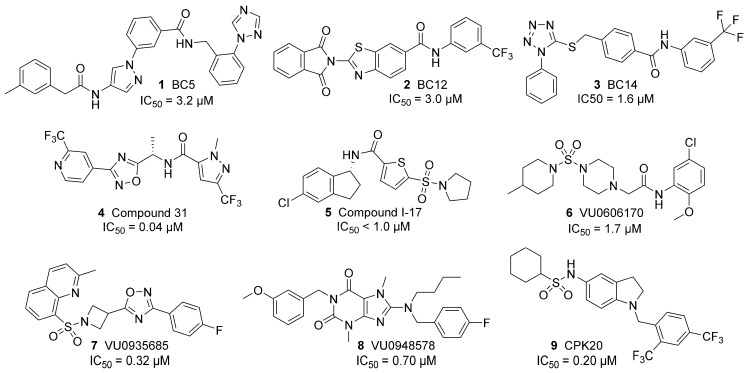
Examples of small molecule SLACK inhibitors that have been reported in the literature. Reported IC_50_ values obtained using whole-cell, patch clamp electrophysiology versus WT SLACK. Refer to original references cited in the text for experimental details, as cell lines and protocols differ.

A handful of older approved drugs have been shown to inhibit SLACK activity in vitro, including quinidine, clofilium, bepridil, and carvedilol; however, their effects in the clinic are quite limited for a variety of reasons. For example, quinidine causes adverse effects as a result of its off-target activities and pharmacokinetic profile [4,8,13,19,29,31,32,33,34,35,36,37,38]. As recently as five years ago, no potent and selective small molecule SLACK inhibitors had been described in the literature. Fortunately, recent progress from a variety of groups has been notable, and inhibitors from multiple distinct chemotypes have now been disclosed (Figure 1) [39]. A variety of strategies have been employed in pursuit of potent and selective SLACK inhibitors, including both high-throughput screening (HTS) and virtual screening approaches. The University of Leeds utilized the latter approach to identify multiple pore blockers of SLACK [40]. Specifically, researchers employed a model based on the structure of the cryo-electron microscopy-derived chicken SLACK protein and a virtual library of 100,000 compounds. This work identified six different compounds that were demonstrated to have micromolar potency versus SLACK in whole-cell, patch clamp assays. While three compounds are classified as pan-assay interference compounds (PAINS) [41,42,43], compounds **1**–**3** (Figure 1) are free of that potential risk. To gauge the potential for cardiovascular safety risk in the scaffolds, these compounds were also screened versus the hERG (K_v_11.1) channel. While compound **1** (BC5) almost completely blocked the channel at 10 µM, the results with compounds **2** (BC12) and **3** (BC14) were less severe—at only 10–20% and 45% blockade, respectively—providing early evidence that SLACK inhibition could be separated from hERG inhibition [40].

Pursuit of novel small molecule SLACK inhibitors has not been limited to academic groups, as evidenced by the multiple publications in the peer-reviewed [44] and patent [45] literature from Praxis Precision Medicine. The discovery of oxadiazole **4** (Figure 1, Compound 31 in the original publication) began with a HTS of approximately 72,000 compounds using a rubidium (^86^Rb)-flux assay versus SLACK mutant P924L. Extensive optimization of the HTS hit identified analog **4**, which was evaluated in automated patch clamp (APC) assays against human and mouse WT SLACK as well as versus a variety of clinically relevant SLACK mutants. The potency of **4** versus human WT SLACK was excellent (IC_50_ = 0.04 µM); however, its activity versus mouse WT SLACK was more modest (IC_50_ = 0.62 µM). Potency values for **4** versus the SLACK mutants also ranged considerably, with the most activity versus G288S (IC_50_ = 0.22 µM) and the least activity versus F364L (IC_50_ = 1.8 µM). Selectivity against a panel of ion channels, including hERG (IC_50_ = 11.9 µM), was good with **4**. Notably, compound **4** displayed low clearance (2.0 mL/min/kg) and complete bioavailability in mice, enabling it to be tested in vivo, and indeed, anticonvulsant effects were observed in *Kcnt1*^L/L^ mice [44]. Multiple patent applications from Praxis have also been published [45], including those describing chemotypes identical and similar to **4** as well as distinct chemotypes. Compound **5** (Figure 1, Compound I-17 in the published patent application) is an example of one such distinct chemotype [46]. While the patent application did not report specific potency values for individual compounds, compound **5** was one of several exemplified analogs reported to have SLACK activity less than one micromolar [46].

Our foray into the SLACK arena began with a HTS of approximately 100,000 compounds using a fluorescence-based thallium (Tl^+^)-flux assay [47] and HEK-293 cells stably expressing WT human SLACK [9]. This campaign yielded several confirmed hits from a variety of chemotypes. Among the HTS hits was 2-amino-*N*-phenylacetamide **6** (Figure 1, VU0606170), which demonstrated an anti-epileptic phenotype in vitro [48] by inhibiting the frequency of calcium (Ca^2+^) oscillations in co-cultured cortical neurons and glial cells [9]. An hERG assay, also employing Tl^+^-flux, showed **6** to be an inhibitor of that channel as well (IC_50_ > 10 µM). Though we thoroughly attempted to develop structure–activity relationships (SAR) for improved potency in this series, these efforts ultimately produced only a few analogs that were equipotent to **6,** as only very minor structural differences were tolerated in this scaffold [15]. Whole-cell APC studies with compound **6** and these related analogs showed the compounds were potent inhibitors of both WT SLACK and the clinically relevant mutant A934T with slightly more activity versus the mutant channel. Turning our attention to a new oxadiazole chemotype proved more fruitful and culminated in the identification of compound **7** (Figure 1, VU0935685) [49]. Compound **7** represents our most potent SLACK inhibitor tool compound to date (APC IC_50_ = 0.32 µM). In addition, **7** was also a potent inhibitor of the A934T mutant with good selectivity against Slo family members SLICK and Maxi-K. Importantly, **7** was inactive at 10 µM versus hERG in our Tl^+^-flux assay for that target [49]. Most recently, we disclosed our optimization efforts in a third series of SLACK inhibitors, leading to the discovery of xanthine **8** (Figure 1, VU0948578) [50]. Analogs in this series proved selective at 10 µM versus SLICK and Maxi-K as well as a host of ion channel counter targets, including hERG. In addition to its submicromolar potency versus human WT SLACK, additional whole-cell APC studies with **8** showed similar potency versus the clinically relevant A934T (IC_50_ = 0.59 µM) and G288S (IC_50_ = 0.71 µM) mutants [50].

Most recently, a collaborative effort across multiple academic institutions in Italy was reported describing their efforts to discover, optimize, and characterize small molecule SLACK inhibitors [51]. These authors initiated their work by building a homology model of human SLACK based on the published structure of the cryo-electron microscopy-derived chicken SLACK protein [52]. Virtual screening of a custom library of more than 800 compounds for binding at the quinidine binding site was used to select 20 chemically diverse hits for functional screening in a manual patch clamp assay. Of these, five compounds proved more than 35-fold more potent as inhibitors of WT SLACK than quinidine. Additional molecular docking, molecular pharmacology, and pharmacokinetic studies were conducted on select analogs. Compound **9** (Figure 1, CPK20) was a potent inhibitor of WT SLACK (IC_50_ = 0.20 µM), WT SLICK (IC_50_ = 1.3 µM), G288S SLACK (IC_50_ = 0.30 µM), and A934T SLACK (IC_50_ = 0.28 µM). Compound **9** also showed no activity on hERG or K_V_7.2 channels. In addition, **9** was shown to be a low clearance compound in a metabolic stability assay using human liver microsomes and was highly bound to plasma proteins.

## 2. Results and Discussion

### 2.1. Selection of Hit Compound VU0545326

**Figure 2:**
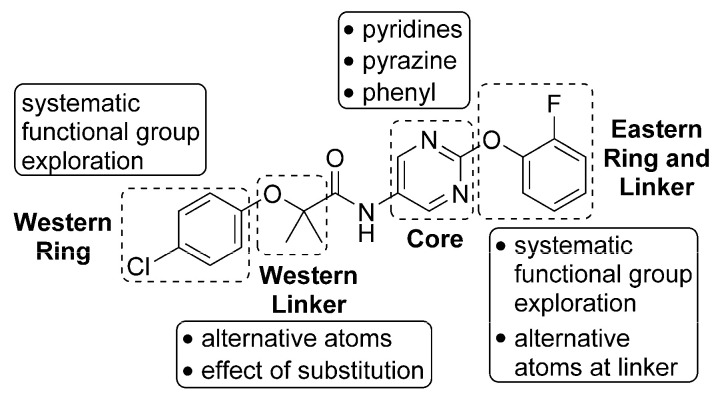
Hit optimization plan for SLACK inhibitor **10** (VU0545326) in four regions.

Despite having made progress in identifying several useful in vitro tools for studying SLACK inhibition [9,15,49,50], we remain interested in pursuing the optimization of additional hits from our HTS campaign. We are convinced that there is value in identifying multiple tools from distinct chemical scaffolds for a variety of reasons. First, observing the same phenotype with SLACK inhibitor tools that are chemically distinct increases our confidence that the effects are indeed driven by SLACK inhibition. Second, possessing chemically distinct SLACK inhibitor tools increases the probability of identifying tools that bind at different binding sites or via different binding modes on SLACK. Finally, we hope to discover a SLACK inhibitor series with properties suitable for a sustained lead optimization effort. Toward that end, pyrimidine HTS hit **10** (VU0545326) was identified as worthy of investigation early in our project (Figure 2). Calculated properties (obtained via PubChem, CID: 71799794) consistent with Lipinski’s guidelines [53] were acceptable: molecular weight (401.8 Da), cLogP (4.3), H-bond donors (1), and H-bond acceptors (6). Values for the topological polar surface area (73.3 Å^2^) and the number of rotatable bonds (6) are also consistent with the potential for good blood-brain barrier (BBB) permeability and oral bioavailability [54,55]. Moreover, the recently published BBB score defines values between 4 and 6 as predictive of good oral absorption and BBB permeability [56]. Determination of the BBB score for **10** gave a value of 4.0. Encouraged by this information, we resynthesized **10** and tested this new batch in our APC assay versus WT SLACK. We were pleased to see good potency (IC_50_ = 1.4 µM) for **10** in this assay and began to design a plan to develop SAR within the scaffold.

Recognizing that we lacked knowledge of the binding site of **10**, which limits the utility of docking and structure-guided drug design approaches, we planned to execute a thorough parallel hit optimization approach in this series. We would endeavor to systematically prepare and test analogs with drug-like functional groups of varying electronic and steric character in four different regions of the scaffold (Figure 2). For example, in the western ring we planned to prepare analogs with such functional groups at each position of the aromatic ring. In the western linker, we planned to look at both the impact of substitution and the nature of the substituent. We also planned to evaluate alternative atoms within the linker. In the core, we planned to look at other six-membered ring aromatic alternatives to the pyrimidine, including pyrazine, phenyl, and pyridines. In the eastern region, we envisioned a similar systematic functional group substitution of the phenyl ring and replacement of the oxygen linker with alternative heteroatoms.

### 2.2. Synthesis of Analogs

**Scheme 1:**
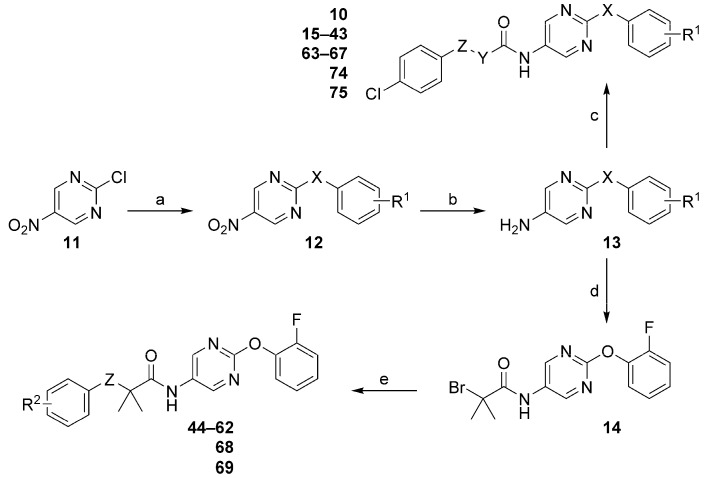
Synthesis of analogs. *Reagents and Conditions:* (**a**) ArXH, K_2_CO_3_, CH_3_CN, µwave, 80 °C, 30 min (For X=NMe, an additional reaction with K_2_CO_3_, MeI, DMF was employed); (**b**) H_2_, Pt(sulfided)/C, MeOH, 70 °C, H-Cube^®^; (**c**) (4-chlorophenyl)-ZYCO_2_H, HATU, DIEA, DMF; (**d**) 2-bromo-2-methylpropionyl bromide, DIEA, CH_2_Cl_2_; (**e**) ArZH, CuBr·SMe_2_, PCy_3_, K_3_PO_4_, CH_3_CN.

Most of our target analogs were easily accessible via one of two short synthetic routes that both began with commercially available 2-chloro-5-nitropyrimidine **11** (Scheme 1). First, reaction of **11** in a nucleophilic aromatic substitution reaction with a host of aryl alcohols, amines, or thiols was used to access intermediates **12**. To access a tertiary amine linker (X=NMe), a subsequent reaction with methyl iodide was employed. Hydrogenation of the nitro group of **12** was accomplished via a poisoned platinum catalyst to provide penultimate amine intermediates **13**. Coupling of **13** with commercial 2-(4-chlorophenoxy)-2-methylpropanoic acid using the hexafluorophosphate azabenzotriazole tetramethyl uranium (HATU) reagent provided the target analogs **10** (X=O), **15**–**43** (X=O), **63** (X=S), **64** (X=NH), and **65**–**67** (X=NMe). Likewise, commercial 3-(4-chlorophenyl)propanoic acid and 2-(4-chlorophenoxy)acetic acid were coupled with **13** using HATU to provide analogs **74** (X=O) and **75** (X=O), respectively. To develop SAR in the western region of the scaffold, we reacted intermediate **13a** (X=O, R=2-F) with 2-bromo-2-methylpropionyl bromide to afford penultimate intermediate **14**. Displacement of the alkyl bromide with aryl alcohols, thiols, and amines was accomplished via copper catalysis [57] to afford target analogs **44**–**62** (Z=O), **68** (X=S), and **69** (X=NH).

**Scheme 2:**
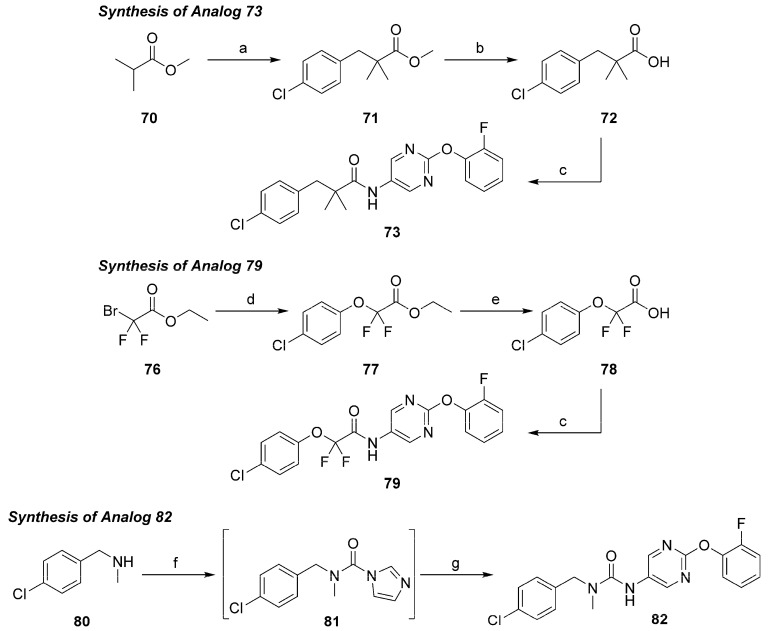
Synthesis of analogs. Reagents and Conditions: (**a**) HN(*i*-Pr)_2_, *n*-BuLi (1.6M in hexanes), THF, −78 °C, then 4-chlorobenzyl bromide, 55%; (**b**) NaOH, MeOH, H_2_O, 60 °C, then 1N aq. HCl, 75%; (**c**) **13a** (X=O, R=2-F), HATU, DIEA, DMF; (**d**) 4-chlorophenol, DBU, DMF, 70 °C; (**e**) NaOH, THF, H_2_O; (**f**) CDI, MeCN, 0 °C to r.t.; (**g**) **13a** (X=O, R=2-F), MeCN, 0 °C to r.t.

Preparation of analogs **73** and **79** required short syntheses of the required carboxylic acids (Scheme 2). For analog **73**, we reacted methyl isobutyrate **70** with lithium diisopropylamide to generate the corresponding lithium enolate, which was reacted in situ with 4-chlorobenzyl bromide to provide intermediate **71**. Basic hydrolysis of **71** afforded acid **72**, which was coupled with intermediate **13a** to give analog **73**. For analog **79**, we alkylated 4-chlorophenol with bromide **76** under basic conditions to generate intermediate **77**. Again, basic hydrolysis provided acid **78**, which was coupled with **13a** to yield analog **79**. To prepare urea analog **82**, we reacted commercial amine **80** with carbonyldiimidazole (CDI) to generate intermediate **81**, which was not isolated but rather reacted with **13a** in situ to afford target analog **82**.

**Scheme 3:**
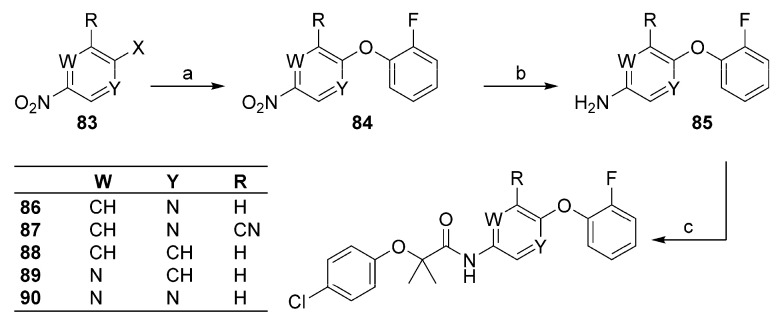
Synthesis of analogs. Reagents and Conditions: (**a**) 2-fluorophenol, K_2_CO_3_, CH_3_CN, µwave, 80 °C, 30 min; (**b**) H_2_, Pt(sulfided)/C, MeOH, 70 °C, H-Cube^®^; (**c**) 3-(4-chlorophenyl)propanoic acid, HATU, DIEA, DMF.

Finally, to prepare analogs that varied in the core of the scaffold, we followed a procedure analogous to that described above in Scheme 1 (Scheme 3). Specifically, we purchased five different halo-nitroarenes **83**: 2-chloro-5-nitropyridine, 2-chloro-5-nitronicotinonitrile, 1-fluoro-4-nitrobenzene, 5-chloro-2-nitropyridine, and 2-chloro-5-nitropyrazine. Each of these monomers was reacted with 2-fluorophenol as described previously to generate the corresponding ether intermediates **84**. Reduction of the nitro group provided the aryl amines **85**, which were subsequently coupled with 2-(4-chlorophenoxy)-2-methylpropanoic acid using HATU to provide the target analogs **86**–**90** as shown.

### 2.3. Structure–Activity Relationships

Using concentrations ranging from 0.125 to 30 µM in half-log steps, a concentration–response relationship was generated for each new analog using our APC assay in CHO-K1 cells stably expressing WT SLACK. A minimum of three cells, i.e., replicates, were collected per concentration for each analog. The eastern ring of the scaffold proved reasonably tolerant to functional group modification (Table 1). Removal of the 2-fluoro group in hit **10** only slightly reduced activity (**15**), and both 3-fluoro (**16**) and 4-fluoro (**17**) substituents were also tolerated with only slight reductions in potency. Similar trends were observed with other electron-withdrawing groups such as chloro (**18**–**20**), trifluoromethyl (**24**–**26**), and trifluoromethoxy (**30**–**32**) where analogs at all positions were within 0.7 µM of the hit **10**. Among these, the 2-chloro (**18**), 4-chloro (**20**), and 4-trifluoromethyl (**26**) analogs showed the most improvement in potency relative to the hit **10**. In the case of methyl (**21**–**23**) and methoxy (**27**–**29**) analogs, none demonstrated superior potency to hit **10**. In the cyano series of analogs (**33**–**35**), we again observed a preference for substitution at the 2- and 4-positions, where both **33** and **35** demonstrated improved activity relative to **10**. Encouraged that a more polar substituent was tolerated in this region, we also evaluated 4-methylsulfone (**36**) and primary carboxamide (**37**) analogs. Interestingly, while sulfone **36** was identical in potency to 4-cyano analog **35**, carboxamide **37** was only a weak inhibitor. Lastly, we chose to evaluate difluoro analogs (**38**–**42**) and found that all analogs except the 3,4-difluoro analog **41** demonstrated equal or superior potency to hit **10**. Among these, 2,6-difluoro analog **40** showed nearly a 3-fold improvement in potency relative to **10**. 2-Chloro-6-fluro analog **43** was similarly potent to **40**.

Unlike the eastern ring region, the western ring proved much less tolerant of modification with a lack of discernable SAR (Table 2). Notably, in this case, the 4-chloro group found in **10** was essential to potent SLACK inhibition, as its deletion found **44** only weakly active. Many of the analogs in this set of compounds were inactive up to the top concentration of 30 µM and no IC_50_ value was determined. These inactive compounds included both lipophilic (**46**–**49**, **51**, **53**, and **56**) and polar (**60** and **61**) substituents. Likewise, 2-fluoro (**45**), 4-trifluoromethoxy (**58**), and 4-cyano (**59**) analogs were only weak inhibitors. Among the six analogs in this set with sufficient potency to enable determination of an IC_50_ value, the most promising results were observed with the 2-methyl (**50**) and 4-trifluoromethyl (**55**) analogs; however, these compounds were still approximately 2-fold less potent than the hit **10**. More moderate inhibition was observed with 4-methyl (**52**), 3-trifluoromethyl (**54**), and 3-trifluoromethoxy (**57**) analogs. The highly electronegative pentafluorosulfanyl group (**62**)—a trifluoromethyl isostere—further reduced activity, perhaps due to its larger volume [58]. While these six analogs were all improved relative to unsubstituted derivative **44**, none represented improvements over the hit **10**.

Having thoroughly evaluated the extremities of the scaffold, we turned our attention to the two linker groups that joined those ring systems to the core (Figure 2). Returning to the eastern region of the scaffold, we prepared and evaluated a handful of analogs that explored replacements for the oxygen atom found in hit compound **10** (Table 3). While it certainly represents a potential metabolic liability, thioether analog **63** did provide a notable improvement in SLACK inhibitory activity. The introduction of a new hydrogen bond donor in the form of amine analog **64** was not tolerated; however, methylation of the nitrogen atom (**65**) nearly restored activity to a comparable level seen with the ether hit compound **10**. Interested in considering some of the more promising eastern ring polar groups in the context of the N-methyl linker, we prepared cyano analog **66** and methyl sulfone analog **67**. Methyl sulfone **67** was only slightly less potent than ether comparator **36**, and the potency of cyano analog **66** was slightly improved relative to ether comparator **35**. In fact, analog **66** provided our fifth compound with submicromolar potency in this series.

Moving to the larger linker that joins the core to the western ring (Figure 2), we focused on variations to the two atoms connecting the ring to the carbonyl carbon of the secondary amide (Table 4). In this case, thioether **68** and secondary amine **69** were approximately 3-fold and 2-fold less potent than hit **10**, respectively. Replacement of the oxygen atom with a methylene (**73**) was less favorable, leading to a 5-fold drop in SLACK inhibitory activity. Interestingly, removing the gem-dimethyl group from **73** to provide analog **74** resulted in a small boost in potency; however, the same change to the hit compound **10** gave analog **75**, which was inactive. On the other hand, replacement of the gem-dimethyl of **10** with a gem-difluoro group (**79**) only resulted in a slight potency reduction. Urea **80** was only a weak inhibitor.

The final region of the chemotype slated for study in our initial foray into this chemotype was the core of the molecule (Table 5). Removal of one of the nitrogen atoms in the pyrimidine core of **10** to yield pyridine analog **86** resulted in roughly a 2-fold loss of activity. Interestingly, most of the lost activity could be restored through introduction of a cyano group at that position in the form of analog **87**. Moving to phenyl core analog **88** saw a further drop in activity—in this case, 3.5-fold less than pyrimidine hit **10** and 1.6-fold less than pyridine **86**. Interestingly, both pyridine analog **89** and pyrazine analog **90** were only weak inhibitors of SLACK, demonstrating that the locations of the nitrogen heteroatom(s) in the core were critical for potency. It may be that introduction of a nitrogen atom adjacent to the carbon serving as the attachment site for the amide portion of the scaffold forces the entire western region of the chemotype into a conformation that is unfavorable for binding to SLACK. Whatever the case may be, our work to date in this region indicates that the pyrimidine core found in the hit compound **10** remains optimal for SLACK activity.

### 2.4. Secondary Pharmacological Profiling

Having executed our plan to prepare analogs across the identified regions of the chemotype (Figure 2), we identified two analogs for further profiling and evaluation. We chose analog **40** (VU0915735) as it was the most potent SLACK inhibitor identified during our studies. Desiring to add a second compound with some measure of structural differences, we also chose another submicromolar SLACK inhibitor **66** (VU0936154). Whereas **40** contains an ether linker and a halogenated phenyl group in the eastern region, **60** contains an *N*-methyl linker and a phenyl group with a more polar cyano substituent. We first screened these analogs at 10 µM versus our in-house panel of ion channel targets using Tl^+^-flux assays in HEK-293 cells expressing the human target of interest [9,59,60]. Our experience with these types of channels has demonstrated that Tl^+^-flux assay results in HEK-293 cells are generally comparable to APC in either HEK-293 or CHO-K1 cells [9,15]. Included in this set are Slo family members SLICK and two isoforms of Maxi-K, as well as several different K^+^, Na^+^, and Ca^2+^ channels (Table 6). Slo family selectivity was encouraging for both compounds, with neither compound approaching 50% of control versus SLICK and Maxi-K. Interestingly, **40** showed weak activation (36% of acetazolamide maximum) versus SLICK, whereas **66** was a weak inhibitor (16% of SKF96365 maximum). Of note was the excellent selectivity versus the broader panel of targets for both compounds, including versus the hERG channel. Thus, we have identified another important example demonstrating that SLACK and hERG inhibition can be separated within a chemotype.

Finally, we chose to screen the same two compounds versus mouse SLACK and two clinically relevant GOF SLACK mutants in APC assays utilizing CHO-K1 cells stably expressing these targets (Table 7). Screening versus the mouse homolog was deemed important for evaluating the potential of the series to ultimately lead to a useful in vivo tool via further optimization [21,61,62,63]. We observed potent inhibition of both mouse WT SLACK and human A934T SLACK that was comparable with previously observed human WT SLACK activity for both **40** and **66**. We were somewhat surprised to find that these compounds were either inactive (**40**) or only weakly active (**66**) versus the G288S mutant. These results stand in stark contrast to our observations with other tool compounds we have profiled, such as VU0606170 (**6**) and VU0948578 (**8**) where the potency for those compounds versus WT, A934T, and G288S were quite similar [9,50]. While additional studies would be required to confirm this hypothesis, it is possible that these results are indicative of a different binding site or binding mode for this pyrimidine series.

### 2.5. Conclusion and Future Directions

In conclusion, we have executed a hit optimization plan across four distinct regions of 2-aryloxy-*N*-(pyrimidin-5-yl)acetamide inhibitors of SLACK potassium channels. More than 60 analogs were prepared and tested using our APC assay in CHO cells stably expressing WT SLACK, leading to the identification of five compounds with submicromolar potency. Substitution was most tolerable in the eastern region of the scaffold whereas the remainder of the scaffold proved more resistant to modifications. Having worked in four distinct SLACK inhibitor scaffolds now [15,49,50], observing narrow SAR in a major portion of the chemotype appears to be common. Future studies to better understand the binding sites of tools identified to date would be quite valuable and help enable future optimization efforts within those scaffolds and beyond. This study has nonetheless identified two related in vitro tools, compounds **40** (VU0915735) and **66** (VU0936154), from a new and distinct chemotype. These analogs both demonstrate good potency for SLACK inhibition for mouse and human WT SLACK as well as the clinically relevant A934T mutant and have selectivity versus other Slo family members and an assortment of diverse ion channel targets including the hERG channel. While **40** and **66** are comparable in potency to many of the previously reported SLACK inhibitor tools (Figure 1), these compounds are from a novel and structurally distinct chemotype. Likewise, **40** and **66** demonstrate preferential activity for WT and the A934T mutant versus the G288S mutant that has yet to be observed by us or reported by others. Next steps for this chemical series include profiling selected analogs using in vitro assays to evaluate their ADME properties and synthesis of additional analogs combining optimal features identified through these and future studies. Results from such studies will be reported in due course.

## 3. Materials and Methods

### 3.1. Synthesis and Purification

Air-sensitive reactions were carried out under a nitrogen atmosphere. Starting materials, reagents, intermediates, and final compounds were weighed on a Mettler Toledo^TM^ (Columbus, OH, USA) New Classic ME analytical balance or a Mettler Toledo^TM^ (Columbus, OH, USA) New Classic ME toploader balance. Thin-layer chromatography (TLC) was conducted on glass plates coated with Silica Gel 60 F_254_ from Millipore Sigma (Burlington, MA, USA). Normal-phase flash chromatography was carried out on either a CombiFlash^®^ EZ Prep or CombiFlash^®^ Rf+ automated flash chromatography system, both from Teledyne ISCO (Lincoln, NE, USA). Normal-phase flash chromatography was carried out using Redi*Sep*^®^ Rf normal-phase, disposable flash columns from Teledyne ISCO (Lincoln, NE, USA) or SiliaSep normal-phase, disposable flash columns (40–63 micron) from SiliCycle, Inc. (Quebec City, QC, CA). Reverse-phase preparative chromatography was carried out on the CombiFlash^®^ EZ Prep using a reusable Redi*Sep*^®^ Rf C18 reverse-phase column. Microwave reactions were carried out with an Anton Paar (Graz, Austria) Monowave 200 automated microwave synthesizer. The Monowave 200 has an output power of 850 W with a maximum temperature of 260 °C and a maximum pressure of 290 psi, and is suitable for use with reaction volumes ranging from 0.5 to 20 mL. Hydrogenation reactions were performed using an H-Cube Mini Plus^TM^ from ThalesNano (Budapest, Hungary).

All NMR spectra were recorded on a 300 MHz Bruker (Billerica, MA, USA) Fourier 300HD NMR spectrometer equipped with a dual ^1^H and ^13^C probe with Z-Gradient and automatic tuning and matching, full computer control of all shims with TopShim^TM^, 24-sample SampleCase^TM^ automation system, and TopSpin^TM^ software (v. 4.2.0). All NMR samples were prepared with either chloroform-d with 0.03% TMS (99.8+ atom % D, Thermo Scientific (Waltham, MA, USA) Catalog No. AC209561000) or methanol-d_4_ with 0.03% TMS (99.8+ atom % D, Thermo Scientific (Waltham, MA, USA) Catalog No. AC351470075). ^1^H and ^13^C chemical shifts are reported in δ values in ppm downfield. Data are reported as follows: chemical shift, multiplicity (s = singlet, d = doublet, t = triplet, q = quartet, br = broad, m = multiplet), integration, coupling constant (Hz). Due to restricted rotation about the amide bond that is present in the final analogs, certain protons (in ^1^H NMR spectra) and carbons (in ^13^C NMR spectra) appear as distinct signals. ^13^C NMR spectra are proton-decoupled but not fluorine-decoupled. Results are reported as they appear from spectra obtained at room temperature. High-resolution mass spectrometry was conducted on an Agilent (Santa Clara, CA, USA) 6230 Accurate-Mass Time-of-Flight (TOF) LC/MS with ESI source equipped with MassHunter Walkup software (v B.09.00, Build 9.09.9044.0). MS parameters were as follows: fragmentor: 175 V, capillary voltage: 3500 V, nebulizer pressure: 35 psig, drying gas flow: 11 L/min, drying gas temperature: 325 °C. Samples were introduced via an Agilent (Santa Clara, CA, USA) 1260 Infinity UHPLC comprised of a G4225A HiP Degasser, G1312B binary pump, G1367E ALS, G1316A TCC, and G1315C DAD VL+ with a 5 μL semi-micro flow cell with a 6 mm path length. UV absorption was observed at 220 nm and 254 nm with a 4 nm bandwidth. Column: Agilent Zorbax SB-C18, Rapid Resolution HT, 1.8 µm, 2.1 × 50 mm. Gradient conditions: Hold at 5% CH_3_CN in H_2_O (0.1% formic acid) for 1.0 min, 5% to 95% CH_3_CN in H_2_O (0.1% formic acid) over 5 min, hold at 95% CH_3_CN in H_2_O (0.1% formic acid) for 1.0 min, 0.5 mL/min. All samples submitted for biological testing were confirmed ≥95% pure by ^1^H NMR.

### 3.2. Analog Synthesis

Synthesis and characterization of analogs **40** (VU0915735) and **66** (VU0936154) are described here as representative of the routes used to access the most active SLACK inhibitor compounds. Characterization of all samples submitted for biological testing are available in the Supplementary Materials.

2-(2,6-Difluorophenoxy)-5-nitropyrimidine (**12b**). 2-Chloro-5-nitropyrimidine (103 mg, 0.646 mmol, 1.0 eq), potassium carbonate (250 mg, 1.614 mmol, 2.5 eq), and 2,6-difluorophenol (146.3 mg, 88.4 µL, 1.162 mmol, 1.8 eq) in anhydrous acetonitrile (2 mL) were dissolved in a G10 microwave vial. The reaction was heated to 80 °C in the microwave reactor for 30 min, and TLC R_f_ = 0.60 (hexanes: ethyl acetate, 1:1) indicated the product was formed. The mixture was diluted with ethyl acetate (5 mL) and hexanes (5 mL) and poured into H_2_O (10 mL). The aqueous layer was extracted with ethyl acetate/hexanes (1:1; 10 mL, 3×). The organic layer was dried over magnesium sulfate and concentrated in vacuo. Purification of the residue by flash chromatography on silica gel yielded 98.1 mg (62%) of the title compound. ^1^H NMR (300 MHz, CDCl_3_) δ 9.35 (s, 2H), 7.34–7.23 (m, 1H), 7.13–7.01 (m, 2H); ^13^C NMR (75 MHz, CDCl_3_) δ 165.60, 156.84, 156.79, 156.46, 153.51, 153.46, 139.69, 127.07, 126.95, 126.83, 112.59, 112.29. LCMS R_T_ = 5.14 min; HRMS, calculated for C_10_H_6_F_2_N_3_O_3_^+^ [M + H], 254.0372; found 254.0368.

2-(2,6-Difluorophenoxy)pyrimidin-5-amine (**13b**). Intermediate **12b** (172 mg, 0.679 mmol) was dissolved in methanol (30 mL) and subjected to hydrogenation using the H-Cube Mini Plus^TM^ (Budapest, Hungary) equipped with a 30 *×* 4 mm 5% Pt/C, sulfided CatCart^®^ (Catalog No. THS 02117, Budapest, Hungary) with a flow rate of 0.5 mL/min at 70 °C. Concentration of the liquid collected from the reactor outflow in vacuo yielded 134 mg (88%) of the title compound. ^1^H NMR (300 MHz, CDCl_3_) δ 8.05 (s, 2H), 7.16 (m, 1H), 7.06–6.95 (m, 2H); ^13^C NMR (75 MHz, CD_3_OD) δ 166.53, 166.48, 165.02, 163.25, 163.19, 153.64, 149.60, 135.56, 135.43, 135.31, 122.21, 122.13, 122.01, 121.93. LCMS R_T_ = 3.99 min; HRMS, calculated for C_10_H_8_F_2_N_3_O^+^ [M + H], 224.0630; found 224.0633.

2-(4-Chlorophenoxy)-*N*-(2-(2,6-difluorophenoxy)pyrimidin-5-yl)-2-methylpropanamide (**40**, VU0915735). In a 50 mL round-bottom flask, 2-(4-chlorophenoxy)-2-methylpropanoic acid (50 mg, 1.0 equiv) was dissolved in DMF. HATU (141 mg, 1.6 equiv.), and DIEA (100 μL, 2.5 equiv) was added and stirred for 10 min. A solution of 2-(2,6-difluorophenoxy)pyrimidin-5-amine (57 mg, 1.1 equiv) in DMF (0.5 mL) was added to the reaction mixture and allowed to stir overnight. Water was added to it and extracted with ethyl acetate (2 × 15 mL). The combined organic layer was washed with water and concentrated in vacuo. Purification of the residue by flash chromatography on silica gel yielded 35 mg (35%) of the title compound. ^1^H NMR (300 MHz, CDCl_3_) δ 8.83 (s, 2H), 8.67 (s, 1H), 7.32–7.23 (m, 2H), 7.23–7.13 (m, 1H), 7.06–6.96 (m, 2H), 6.95–6.86 (m, 2H), 1.56 (s, 6H); ^13^C NMR (75 MHz, CDCl_3_) δ 173.17, 160.37, 157.41, 157.36, 154.09, 154.03, 151.32, 129.69, 129.55, 129.29, 126.00, 125.88, 125.76, 123.57, 112.39, 112.09, 82.36, 24.76. LCMS R_T_ = 5.68 min; HRMS, calculated for C_20_H_17_ClF_2_N_3_O_3_^+^ [M + H], 420.0921; found 420.0925.

4-(Methyl(5-nitropyrimidin-2-yl)amino)benzonitrile (**12c**). (i) In a G30 microwave vial, 2-chloro-5-nitropyrimidine (300 mg, 1.88 mmol, 1.3 eq), potassium carbonate (500 mg, 3.62 mmol, 2.5 eq), and 4-aminobenzonitrile (171 mg, 1.45 mmol, 1.0 eq) in acetonitrile (5 mL) were dissolved. The reaction was heated to 80 °C in the microwave reactor for 45 min. TLC indicated the starting material was consumed completely. The reaction mixture was diluted with ethyl acetate (10 mL). The aqueous layer was extracted with hexanes: ethyl acetate (1:1), and the organic layers were washed with a 10% HCl (aq) solution. The combined organic layers were then washed with sodium bicarbonate (10 mL × 3) and brine (10 mL × 3). The solution was dried over sodium sulfate, filtered, and concentrated in vacuo. Purification of the residue by flash chromatography on silica gel yielded 129 mg (37%) of 4-((5-nitropyrimidin-2-yl)amino)benzonitrile. ^1^H NMR (300 MHz, CD_3_OD) δ 11.25 (br s, 1H), 9.33 (s, 2H), 8.01 (d, *J* = 8.8 Hz, 2H), 7.85 (d, *J* = 8.8 Hz, 2H); ^13^C NMR (75 MHz, CD_3_OD) δ 170.18, 164.63, 152.45, 145.61, 143.25, 142.71, 129.60, 114.58. LCMS R_T_ = 4.37 min; HRMS, calculated for C_11_H_8_N_5_O_2_^+^ [M + H], 242.0673; found 242.0671. (ii) 4-((5-Nitropyrimidin-2-yl)amino)benzonitrile (66 mg, 0.27 mmol, 1.0 eq), potassium carbonate (267 mg, 1.93 mmol, 7.2 eq) and methyl iodide (45 µL, 0.72 mmol, 2.7 eq) were dissolved in DMF (4 mL). The reaction was monitored by TLC to completion and diluted with water and ethyl acetate. The layers were separated, and the aqueous layer was extracted with ethyl acetate. The combined organics were washed with a 10% NaOH (aq) solution and concentrated in vacuo. Purification of the residue by flash chromatography on silica gel yielded 15.8 mg (23%) of the title compound. ^1^H NMR (300 MHz, CDCl_3_) δ 9.12 (s, 2H), 7.76 (d, *J* = 8.5 Hz, 2H), 7.48 (d, *J* = 8.5 Hz, 2H), 3.68 (s, 3H); ^13^C NMR (75 MHz, CDCl_3_) δ 161.99, 154.63, 147.58, 135.45, 133.29, 127.05, 118.26, 110.64, 39.32. LCMS R_T_ = 4.97 min; HRMS, calculated for C_12_H_10_N_5_O_2_^+^ [M + H], 256.0829; found 256.0830.

4-((5-Aminopyrimidin-2-yl)(methyl)amino)benzonitrile (**13c**). Intermediate **12c** (15.8 mg, 0.0619 mmol) was dissolved in methanol (30 mL) and subjected to hydrogenation using the H-Cube Mini Plus^TM^ (Budapest, Hungary) equipped with a 30 *×* 4 mm 5% Pt/C, sulfided CatCart^®^ (Catalog No. THS 02117, Budapest, Hungary) with a flow rate of 1.0 mL/min at 70 °C. The liquid collected from the reactor outflow was concentrated in vacuo, and the residue was purified using flash chromatography on silica gel to yield 4.2 mg (30%) of the title compound. ^1^H NMR (300 MHz, CDCl_3_) δ 8.04 (s, 2H), 7.59 (d, *J* = 8.8 Hz, 2H), 7.41 (d, *J* = 8.8 Hz, 2H), 3.57 (s, 3H), 3.41 (br s, 2H). LCMS R_T_ = 3.64 min; HRMS, calculated for C_12_H_12_N_5_^+^ [M + H], 226.1087; found 226.1087.

2-(4-Chlorophenoxy)-*N*-(2-((4-cyanophenyl)(methyl)amino)pyrimidin-5-yl)-2-methylpropanamide (**66**, VU0936154). In a 25 mL round-bottom flask, 2-(4-chlorophenoxy)-2-methylpropanoic acid (29 mg, 1.0 equiv) was dissolved in DMF. HATU (81 mg, 1.6 equiv.) and DIEA (60 μL, 2.5 equiv) was added and stirred for 10 min. A solution of 4-((5-aminopyrimidin-2-yl)(methyl)amino)benzonitrile (28 mg, 1 equiv) in DMF (0.5 mL) was added to the reaction mixture and allowed to stir overnight. Water was added to it and extracted with ethyl acetate (2 *×* 15 mL). The combined organic layer was washed with water and concentrated under reduced pressure. Purification of the residue by flash chromatography on silica gel yielded 25 mg (43%) of the title compound. ^1^H NMR (300 MHz, CDCl_3_) δ 8.63 (s, 2H), 8.36 (s, 1H), 7.76–7.58 (m, 2H), 7.52–7.39 (m, 2H), 7.35–7.21 (m, 2H), 7.01–6.83 (m, 2H), 3.61 (s, 3H), 1.57 (s, 6H); ^13^C NMR (75 MHz, CDCl_3_) δ 173.09, 158.35, 152.11, 150.44, 149.22, 132.78, 129.55, 125.03, 124.99, 123.35, 119.04, 107.35, 82.33, 38.10, 24.85. LCMS R_T_ = 5.88 min; HRMS, calculated for C_22_H_21_ClN_5_O_2_^+^ [M + H], 422.1378; found 422.1382.

### 3.3. Stable Cell Line Generation

Stably transfected monoclonal HEK293 cell lines expressing SLICK, Maxi-K α1/β3, Maxi-K α1/β4, Ca_V_3.2, GIRK 1/2, K_V_2.1, K_V_7.2, Na_V_1.7, and TASK-1 were generated as previously described [9]. Stably transfected monoclonal HEK293 cell lines expressing hERG were generated as previously described [59]. CHO-K1 cells expressing human SLACK and disease-associated human SLACK mutations, G288S and A934T, were generated by transfecting a PiggyBac TRE mammalian expression vector (VectorBuilder, Chicago, IL, USA) containing KCNT1. FuGENE 6 (Promega, Madison, WI, USA) transfection reagent and protocol, together with Opti-MEM (Thermo Fisher Scientific, Waltham, MA, USA), were utilized according to the manufacturer’s instructions.

### 3.4. Whole-Cell Automated Patch Clamp Electrophysiology

Automated patch clamp recording was performed with a SyncroPatch 768 PE system (Nanion, Munich, Germany) as previously described [9,64]. Whole-cell recordings were performed at room temperature. Cells were harvested by first washing with PBS without Mg^2+^ or Ca^2+^ (Gibco, Billings, MT, USA) to remove cell culture medium, followed by dissociation with TryPLE (Gibco, Billings, MT, USA). Cells were then resuspended and diluted to 200,000 cells/mL in external solution. External solution contained 105 mM NaCl, 4 mM KCl, 1 mM MgCl_2_, 5 mM CaCl_2_, 10 mM HEPES, 40 mM N-Methyl-D-glucamine chloride, titrated to pH 7.4 with NaOH and osmolarity of 300 mOsm/kg; internal solution contained 70 mM NaCl, 70 mM KF, 10 mM KCl, 5 mM EGTA, 5mM HEPES, titrated to a pH 7.2 with KOH and osmolarity of 295 mOsm/kg. Compound potencies were generated using concentrations ranging from 0.125 to 30 μM in half-log steps and a minimum of three cells (replicates) per concentration series.

The APC protocol was set as following: Cells were held at a pressure of −80 mV for 100 ms, then a ramp protocol was performed from −100 mV to 80 mV at 0.4 mV/ms, hold the cell at −80 mV for another 100 ms, then steps were performed at −20 mV, 0 mV, +20 mV, +40 mV, and +60 mV for 100 ms of each step. Whole-cell currents were measured at 0 mV and normalized to current after external buffer addition. Compound response compared to “full block” E_max_ of 500 μM quinidine. To obtain the concentration-response relationship graphs and IC_50_ values, ΔA values for each compound were taken from the median value of the last 7 current ampliutude values obtained for each set of data points collected for a given concentration at a particular voltage step; these values were used to generate fits to a four-parameter variable slope nonlinear regression model using GraphPad Prism 9 (GraphPad Prism Software, San Diego, CA, USA); potencies given as mean (95% CI).

### 3.5. Thallium (Tl^+^) Flux Assays

The Tl^+^ flux assays were conducted as previously described [9,60], with modifications optimized for individual cell lines as described below. Following removal of cell culture media, cells were loaded with Tl^+^-sensitive dye loading solution containing Thallos-AM (ION Biosciences, San Marcos, TX, USA), 1.25 mM Probenecid and 0.3% Pluronic F-127 (MilliporeSigma, Burlington, MA, USA), in assay buffer, comprised of Hanks Balanced Salt Solution (HBSS) (Thermo Fisher Scientific, Waltham, MA, USA) and 20 mM HEPES (Corning, Corning, NY, USA), titrated to pH 7.3 with NaOH in at room temperature for one-hour. The dye loading solution was replaced with 20 μL/well of assay buffer prior to assaying. Cell plates were then loaded onto a Panoptic kinetic imaging plate reader (WaveFront Biosciences, Franklin, TN, USA). Data were acquired at 5 Hz (excitation 482 ± 35 nm, emission 536 ± 40 nm) for 10 s, at which time 20 μL/well of test compounds in assay buffer at 2-fold above the desired final concentration was added and allowed to incubate for 120 s. Next, 10 μL/well of Tl^+^ stimulus buffer (129 mM Na gluconate, 1.2 mM CaSO_4_, 1 MgSO_4_, 1 mM Na_2_HPO_4_, 4.17 mM NaHCO_3_, 5.56 mM glucose, 2.5 mM Tl_2_SO_4_, and 10 mM HEPES, titrated to pH 7.3 with NaOH) was added. Imaging concluded after an additional 120 s. Compound potencies were determined using concentrations ranging from 1.5 nm to 30 μM in half-log steps and a minimum of three replicate wells per concentration series.

## Figures and Tables

**Table 1:**
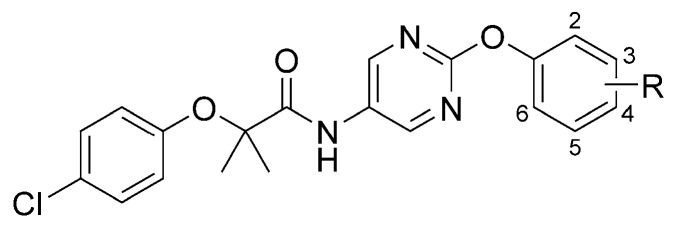
Eastern ring analogs.

Analog	R	SLACK IC_50_ (µM) ^1^	SLACK I_max_ ^1,2^
**10**	2-F	1.4	100
**15**	H	1.7	100
**16**	3-F	2.1	100
**17**	4-F	1.8	102
**18**	2-Cl	1.2	97
**19**	3-Cl	1.5	100
**20**	4-Cl	1.2	100
**21**	2-Me	2.9	100
**22**	3-Me	2.3	100
**23**	4-Me	1.7	100
**24**	2-CF_3_	1.7	100
**25**	3-CF_3_	1.9	101
**26**	4-CF_3_	1.0	100
**27**	2-OMe	1.5	101
**28**	3-OMe	2.4	101
**29**	4-OMe	1.8	100
**30**	2-OCF_3_	1.4	100
**31**	3-OCF_3_	1.3	100
**32**	4-OCF_3_	1.9	100
**33**	2-CN	0.94	100
**34**	3-CN	1.7	100
**35**	4-CN	1.1	100
**36**	4-SO_2_Me	1.1	100
**37**	4-CONH_2_	>10	110
**38**	2,4-di-F	1.0	101
**39**	2,5-di-F	1.3	101
**40**	2,6-di-F	0.52	98
**41**	3,4-di-F	1.7	100
**42**	3,5-di-F	1.4	100
**43**	2-Cl, 6-F	0.58	91

^1^ Concentration–response relationships obtained from APC (SyncroPatch 384 from Nanion Technologies GmbH) in CHO-K1 cells stably expressing WT human SLACK. Values represent averages (n ≥ 3). ^2^ Maximum inhibition at 30 µM test compound standardized to VU0606170 (set at 100%).

**Table 2:**
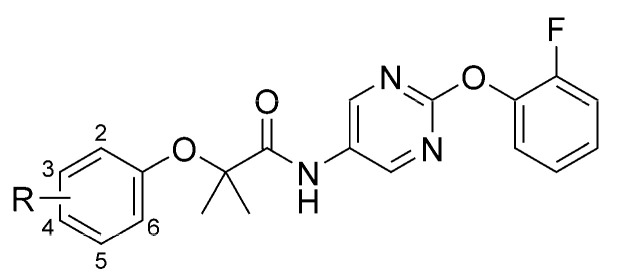
Western ring analogs.

Analog	R	SLACK IC_50_ (µM) ^1^	SLACK I_max_ ^1,2^
**44**	H	>10	66
**45**	2-F	>10	25
**46**	3-F	Not Determined	
**47**	4-F	Not Determined	
**48**	2-Cl	Not Determined	
**49**	3-Cl	Not Determined	
**50**	2-Me	2.9	100
**51**	3-Me	Not Determined	
**52**	4-Me	7.1	97
**53**	2-CF_3_	Not Determined	
**54**	3-CF_3_	3.7	99
**55**	4-CF_3_	2.8	101
**56**	2-OCF_3_	Not Determined	
**57**	3-OCF_3_	4.6	98
**58**	4-OCF_3_	>10	104
**59**	4-CN	>10	109
**60**	4-SO_2_Me	Not Determined	
**61**	4-CONH_2_	Not Determined	
**62**	4-SF_5_	4.1	100

^1^ Concentration–response relationships obtained from APC (SyncroPatch 384 from Nanion Technologies GmbH, Berlin, Germany) in CHO-K1 cells stably expressing WT human SLACK. Values represent averages (n ≥ 3). ^2^ Maximum inhibition at 30 µM test compound standardized to VU0606170 (set at 100%).

**Table 3:**
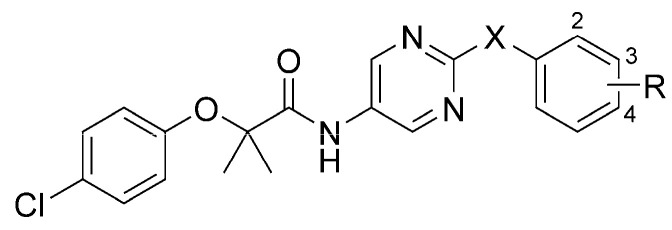
Eastern linker analogs.

Analog	X	R	SLACK IC_50_ (µM) ^1^	SLACK I_max_ ^1,2^
**63**	S	2-F	0.89	101
**64**	NH	2-F	Not Determined	
**65**	NMe	2-F	2.1	100
**66**	NMe	4-CN	0.91	95
**67**	NMe	4-SO_2_Me	1.4	101

^1^ Concentration–response relationships obtained from APC (SyncroPatch 384 from Nanion Technologies GmbH) in CHO-K1 cells stably expressing WT human SLACK. Values represent averages (n ≥ 3). ^2^ Maximum inhibition at 30 µM test compound standardized to VU0606170 (set at 100%).

**Table 4:**
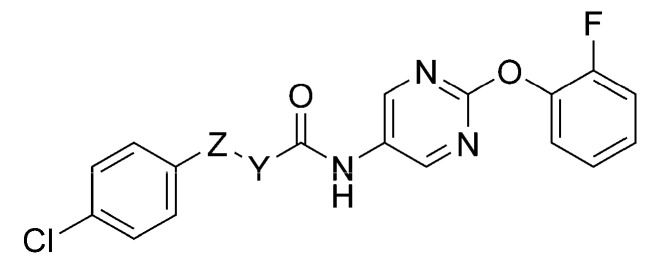
Western linker analogs.

Analog	Z	Y	SLACK IC_50_ (µM) ^1^	SLACK I_max_ ^1,2^
**68**	S	CMe_2_	4.1	100
**69**	NH	CMe_2_	2.7	100
**73**	CH_2_	CMe_2_	7.2	100
**74**	CH_2_	CH_2_	5.1	102
**75**	O	CH_2_	Not Determined	
**79**	O	CF_2_	2.1	100
**82**	CH_2_	NMe	>10	79

^1^ Concentration–response relationships obtained from APC (SyncroPatch 384 from Nanion Technologies GmbH) in CHO-K1 cells stably expressing WT human SLACK. Values represent averages (n ≥ 3). ^2^ Maximum inhibition at 30 µM test compound standardized to VU0606170 (set at 100%).

**Table 5:**
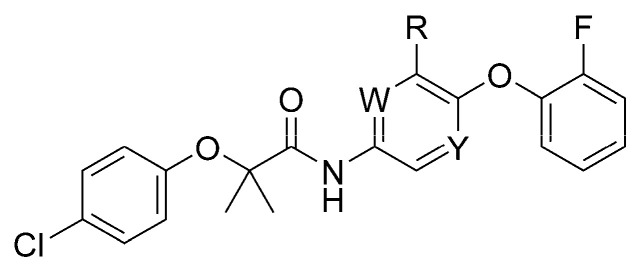
Core analogs.

Analog	W	Y	R	SLACK IC_50_ (µM) ^1^	SLACK I_max_ ^1,2^
**86**	CH	N	H	3.1	100
**87**	CH	N	CN	1.8	100
**88**	CH	CH	H	4.9	92
**89**	N	CH	H	>10	52
**90**	N	N	H	>10	50

^1^ Concentration–response relationships obtained from APC (SyncroPatch 384 from Nanion Technologies GmbH) in CHO-K1 cells stably expressing WT human SLACK. Values represent averages (n ≥ 3). ^2^ Maximum inhibition at 30 µM test compound standardized to VU0606170 (set at 100%).

**Table 6:**
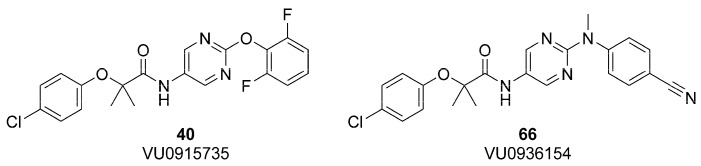
Ion channel panel screening with **40** (VU0915735) and **66** (VU0936154) ^1^.

	Percent Inhibition ± SEM ^2^	
Target	40 (VU0915735)	66 (VU0936154)
SLICK	36 ± 8.8 (activator) ^3^	16 ± 1.4
Maxi-K α1/β3	27 ± 11	30 ± 2.6
Maxi-K α1/β4	10 ± 7.4	22 ± 2.5
Ca_V_3.2	10 ± 0.82	32 ± 2.4
GIRK 1/2	6.8 ± 3.3	6.4 ± 1.5
hERG	0.90 ± 2.9	13 ± 3.3
K_V_2.1	13 ± 5.0	10 ± 1.6
K_V_7.2	1.3 ± 1.0	1.6 ± 0.94
Na_V_1.7	7.5 ±1.7	17 ± 1.8
TASK-1	24 ± 3.7	18 ± 2.0

^1^ Obtained with 10 µM test compound via Tl^+^-flux assay in HEK-293 cells stably expressing target. ^2^ Percent inhibition relative to the following control compounds: SKF96365 (SLICK), paxilline (Maxi-K), bepridil (Ca_V_3.2, hERG, TASK1), SCH23390 (GIRK 1/2), guangxitoxin 1E (K_V_2.1), XE991 (K_V_7.2), and TC-N 1752 (Na_V_1.7). ^3^ Percent activation relative to the following control compound: acetazolamide.

**Table 7:**
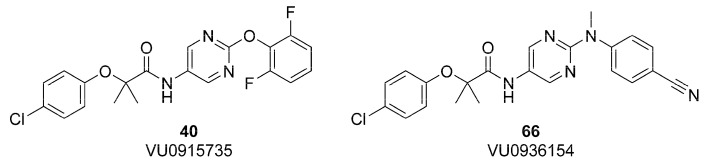
Electrophysiology results versus mouse and GOF mutant SLACK.

Analog	Target	IC_50_ (µM) ^1^	I_max_ ^1,2^
	Mouse WT	0.32	100
**40**	Human A934T	0.41	96
	Human G288S	Not Determined	
	Mouse WT	0.79	91
**66**	Human A934T	1.2	84
	Human G288S	>10	40

^1^ Concentration–response relationships obtained from APC (SyncroPatch 384 from Nanion Technologies GmbH) in CHO-K1 cells stably expressing target. ^2^ Maximum inhibition at 30 µM test compound standardized to VU0606170 (set at 100%).

## Data Availability

Data supporting reported results are available upon request.

## Supplementary Materials

The following supporting information can be downloaded at: https://www.mdpi.com/article/10.3390/molecules29235494/s1, characterization of all samples submitted for biological testing.
