# Supplementary material for: Structure–Activity Relationship Studies in a Series of 2-Aryloxy-N-(pyrimidin-5-yl)acetamide Inhibitors of SLACK Potassium Channels
Source: Molecules. 2024 Nov 21;29(23):5494. doi: 10.3390/molecules29235494 (PMC11643494; doi:10.3390/molecules29235494)
Supplement: Supplementary file 1 [file molecules-29-05494-s001.zip › molecules-3318986-supplementary.pdf]

Structure-Activity Relationship Studies in a Series of 2-Aryloxy-*N*-(pyrimidin-5-yl)acetamide Inhibitors of SLACK Potassium Channels

Nigam M. Mishra [1], Brittany D. Spitznagel [3], Yu Du [3,4], Yasmeen K. Mohamed [1], Ying Qin [1,2], C. David Weaver [3,4], and Kyle A. Emmitte [1,*]

[1] Department of Pharmaceutical Sciences, UNT System College of Pharmacy, University of North Texas Health Science Center, Fort Worth, TX 76107 USA

[2] College of Biomedical and Translational Sciences, University of North Texas Health Science Center, Fort Worth, TX 76107 USA

[3] Department of Pharmacology, Vanderbilt University, Nashville, TN 37232 USA

[4] Vanderbilt Institute for Chemical Biology, Vanderbilt University, Nashville, TN 37232 USA

* Correspondence: kyle.emmitte@unthsc.edu; Tel.: +1 817 735 0241

Synthesis and Purification

Air-sensitive reactions were carried out under a nitrogen atmosphere. Starting materials, reagents, intermediates, and final compounds were weighed on a Mettler Toledo™ New Classic ME analytical balance or a Mettler Toledo™ New Classic ME toploader balance. Thin-layer chromatography (TLC) was conducted on glass plates coated with Silica Gel 60 F_{254} from Millipore Sigma. Normal-phase flash chromatography was carried out on either a CombiFlash® EZ Prep or CombiFlash® Rf+ automated flash chromatography system, both from Teledyne ISCO. Normal-phase flash chromatography was carried out using Redi*Sep*® Rf normal-phase, disposable flash columns from Teledyne ISCO or SiliaSep normal-phase, disposable flash columns (40-63 micron) from SiliCycle, Inc. Reverse-phase preparative chromatography was carried out on the CombiFlash® EZ Prep using a reusable Redi*Sep*® Rf C18 reverse-phase column. Microwave reactions were carried out an Anton Paar Monowave 200 automated microwave synthesizer. The Monowave 200 has an output power of 850W with a maximum temperature of 260 °C and a maximum pressure of 290 psi and is suitable for use with reaction volumes ranging from 0.5 to 20 mL.

All NMR spectra were recorded on a 300 MHz Bruker Fourier 300HD NMR spectrometer equipped with a dual ^{1}H and ^{13}C probe with Z-Gradient and automatic tuning and matching, full computer control of all shims with TopShim™, 24-sample SampleCase™ automation system, and TopSpin™ software. All NMR samples were prepared with chloroform-d with 0.03% TMS (99.8+ atom % D, Thermo Scientific Catalog No. AC209561000) or methanol-d_4 with 0.03% TMS (99.8+ atom % D, Thermo Scientific Catalog No. AC351470075). ^{1}H and ^{13}C chemical shifts are reported in δ values in ppm downfield. Data are reported as follows: chemical shift, multiplicity (s = singlet, d = doublet, t = triplet, q = quartet, br = broad, m = multiplet), integration, coupling constant (Hz). High resolution mass spectrometry was conducted on an Agilent 6230 Accurate-

Mass Time-of-Flight (TOF) LC/MS with ESI source equipped with MassHunter Walkup software. MS parameters were as follows: fragmentor: 175 V, capillary voltage: 3500 V, nebulizer pressure: 35 psig, drying gas flow: 11 L/min, drying gas temperature: 325 °C. Samples were introduced via an Agilent 1260 Infinity UHPLC comprised of a G4225A HiP Degasser, G1312B binary pump, G1367E ALS, G1316A TCC, and G1315C DAD VL+ with a 5 μL semi-micro flow cell with a 6 mm path length. UV absorption was observed at 220 nm and 254 nm with a 4 nm bandwidth. Column: Agilent Zorbax SB-C18, Rapid Resolution HT, 1.8 μm, 2.1 x 50 mm. Gradient conditions: Hold at 5% CH_3CN in H_2O (0.1% formic acid) for 1.0 min, 5% to 95% CH_3CN in H_2O (0.1% formic acid) over 5 min, hold at 95% CH_3CN in H_2O (0.1% formic acid) for 1.0 min, 0.5 mL/min. All samples submitted for biological testing were confirmed ≥ 95% pure by ^{1}H NMR.

NOTE: Due to restricted rotation about the amide bond that is present in the final analogs, certain protons (in ^{1}H NMR spectra) and carbons (in ^{13}C NMR spectra) appear as distinct signals. ^{13}C NMR spectra are proton-decoupled but not fluorine-decoupled. Results are reported as they appear from spectra obtained at room temperature.

Synthesis and Characterization of Analog 40 (VU0915735)

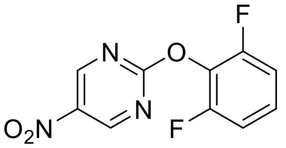

2-(2,6-Difluorophenoxy)-5-nitropyrimidine (12b). 2-Chloro-5-nitropyrimidine (103 mg, 0.646 mmol, 1.0 eq), potassium carbonate (250 mg, 1.614 mmol, 2.5 eq), and 2,6-difluorophenol (146.3 mg, 88.4 μL, 1.162 mmol, 1.8 eq) in anhydrous acetonitrile (2 mL) were dissolved in a G10 microwave vial. The reaction was heated to 80 °C in the microwave reactor for 30 minutes, and TLC R_f = 0.60 (hexanes: ethyl acetate, 1:1) indicated the product was formed. The mixture was diluted with ethyl acetate (5 mL) and hexanes

(5 mL) and poured into H_2O (10 mL). The aqueous layer was extracted with ethyl acetate/hexanes (1:1; 10 mL, 3x). The organic layer was dried over magnesium sulfate and concentrated *in vacuo*. Purification of the residue by flash chromatography on silica gel yielded 98.1 mg (62%) of the title compound. ^{1}H NMR (300 MHz, $CDCl_3$) δ 9.35 (s, 2H), 7.34 – 7.23 (m, 1H), 7.13 – 7.01 (m, 2H); ^{13}C NMR (75 MHz, $CDCl_3$) δ 165.60, 156.84, 156.79, 156.46, 153.51, 153.46, 139.69, 127.07, 126.95, 126.83, 112.59, 112.29. LCMS R_T = 5.14 min; HRMS, calc'd for $C_{10}H_6F_2N_3O_3^+$ [M+H], 254.0372; found 254.0368.

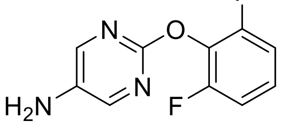

2-(2,6-Difluorophenoxy)pyrimidin-5-amine (13b). Intermediate **12b** (172 mg, 0.679 mmol) was dissolved in methanol (30 mL) and subjected to hydrogenation using the H-Cube Mini Plus™ equipped with a 30x4 mm 5% Pt/C, sulfided CatCart® (Catalog No. THS 02117) with a flow rate of 0.5 mL/min at 70 ºC. Concentration of the liquid collected from the reactor outflow *in vacuo* yielded 134 mg (88%) of the title compound. ^{1}H NMR (300 MHz, $CDCl_3$) δ 8.05 (s, 2H), 7.16 (m, 1H), 7.06-6.95 (m, 2H); ^{13}C NMR (75 MHz, CD_3OD) δ 166.53, 166.48, 165.02, 163.25, 163.19, 153.64, 149.60, 135.56, 135.43, 135.31, 122.21, 122.13, 122.01, 121.93. LCMS R_T = 3.99 min; HRMS, calc'd for $C_{10}H_8F_2N_3O^+$ [M+H], 224.0630; found 224.0633.

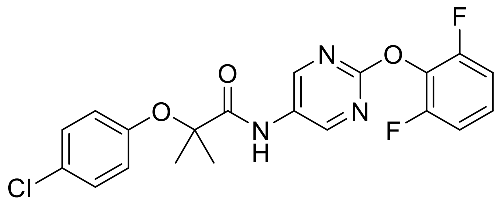

2-(4-Chlorophenoxy)-*N*-(2-(2,6-difluorophenoxy)pyrimidin-5-yl)-2-methylpropanamide (40, VU0915735). In a 50 mL round-bottom flask, 2-(4-

chlorophenoxy)-2-methylpropanoic acid (50 mg, 1.0 equiv) was dissolved in DMF. HATU (141 mg, 1.6 equiv.) and DIEA (100 uL, 2.5 equiv) was added and stirred for 10 mins. A solution of 2-(2,6-difluorophenoxy)pyrimidin-5-amine (57 mg, 1.1 equiv) in DMF (0.5 mL) was added to the reaction mixture and allowed to stir overnight. Water was added to it and extracted with ethyl acetate (2 x 15 mL). The combined organic layer was washed with water and concentrated *in vacuo*. Purification of the residue by flash chromatography on silica gel yielded 35 mg (35%) of the title compound. ^{1}H NMR (300 MHz, $CDCl_3$) δ 8.83 (s, 2H), 8.67 (s, 1H),7.32 – 7.23 (m, 2H), 7.23 – 7.13 (m, 1H), 7.06 – 6.96 (m, 2H), 6.95 – 6.86 (m, 2H), 1.56 (s, 6H); ^{13}C NMR (75 MHz, $CDCl_3$) δ 173.17, 160.37, 157.41, 157.36, 154.09, 154.03, 151.32, 129.69, 129.55, 129.29, 126.00, 125.88, 125.76, 123.57, 112.39, 112.09, 82.36, 24.76. LCMS R_T = 5.68 min; HRMS, calc'd for $C_{20}H_{17}ClF_2N_3O_3^+$ [M+H], 420.0921; found 420.0925.

Synthesis and Characterization of Analog 66 (VU0936154)

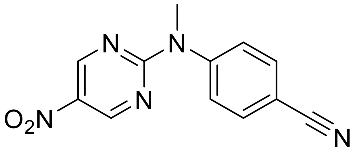

4-(Methyl(5-nitropyrimidin-2-yl)amino)benzonitrile (12c). (*i*) In a G30 microwave vial was dissolved 2-chloro-5-nitropyrimidine (300 mg, 1.88 mmol, 1.3 eq), potassium carbonate (500 mg, 3.62 mmol, 2.5 eq), and 4-aminobenzonitrile (171 mg, 1.45 mmol, 1.0 eq) in acetonitrile (5 mL). The reaction was heated to 80 °C in the microwave reactor for 45 minutes. TLC indicated the starting material was consumed completely. The reaction mixture was diluted with ethyl acetate (10 mL). The aqueous layer was extracted with hexanes: ethyl acetate (1:1), and the organic layers were washed with a 10% HCl (aq) solution. The combined organic layers were then washed with sodium bicarbonate (10 mL x 3) and brine (10 mL x 3). The solution was dried over sodium sulfate, filtered, and concentrated *in vacuo*. Purification of the residue by flash chromatography on silica gel

yielded 129 mg (37%) of 4-((5-nitropyrimidin-2-yl)amino)benzonitrile. ^{1}H NMR (300 MHz, CD_3OD) δ 11.25 (br s, 1H), 9.33 (s, 2H), 8.01 (d, J = 8.8 Hz, 2H), 7.85 (d, J = 8.8 Hz, 2H); ^{13}C NMR (75 MHz, CD_3OD) δ 170.18, 164.63, 152.45, 145.61, 143.25, 142.71, 129.60, 114.58. LCMS R_T = 4.37 min; HRMS, calc'd for $C_{11}H_8N_5O_2^+$ [M+H], 242.0673; found 242.0671. (*ii*) 4-((5-Nitropyrimidin-2-yl)amino)benzonitrile (66 mg, 0.27 mmol, 1.0 eq), potassium carbonate (267 mg, 1.93 mmol, 7.2 eq) and methyl iodide (45 µL, 0.72 mmol, 2.7 eq) were dissolved in DMF (4 mL). The reaction was monitored by TLC to completion and diluted with water and ethyl acetate. The layers were separated, and the aqueous layer was extracted with ethyl acetate. The combined organics were washed with a 10% NaOH (aq) solution and concentrated *in vacuo*. Purification of the residue by flash chromatography on silica gel yielded 15.8 mg (23%) of the title compound. ^{1}H NMR (300 MHz, $CDCl_3$) δ 9.12 (s, 2H), 7.76 (d, J = 8.5 Hz, 2H), 7.48 (d, J = 8.5 Hz, 2H), 3.68 (s, 3H); ^{13}C NMR (75 MHz, $CDCl_3$) δ 161.99, 154.63, 147.58, 135.45, 133.29, 127.05, 118.26, 110.64, 39.32. LCMS R_T = 4.97 min; HRMS, calc'd for $C_{12}H_{10}N_5O_2^+$ [M+H], 256.0829; found 256.0830.

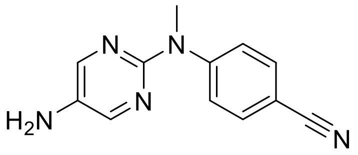

4-((5-Aminopyrimidin-2-yl)(methyl)amino)benzonitrile (13c). Intermediate **12c** (15.8 mg, 0.0619 mmol) was dissolved in methanol (30 mL) and subjected to hydrogenation using the H-Cube Mini Plus™ equipped with a 30x4 mm 5% Pt/C, sulfided CatCart® (Catalog No. THS 02117) with a flow rate of 0.5 mL/min at 70 ºC. The liquid collected from the reactor outflow was concentrated *in* vacuo, and the residue was purified using flash chromatography on silica gel to yield 4.2 mg (30%) of the title compound. ^{1}H NMR (300 MHz, $CDCl_3$) δ 8.04 (s, 2H), 7.59 (d, J = 8.8 Hz, 2H), 7.41 (d, J = 8.8 Hz, 2H), 3.57 (s,

3H), 3.41 (br s, 2H). LCMS R_T = 3.64 min; HRMS, calc'd for $C_{12}H_{12}N_5^+$ [M+H], 226.1087; found 226.1087.

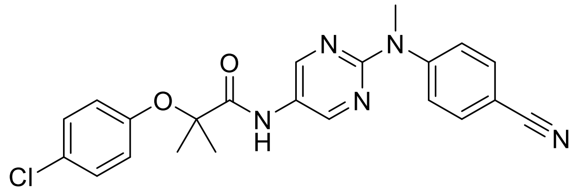

2-(4-Chlorophenoxy)-*N*-(2-((4-cyanophenyl)(methyl)amino)pyrimidin-5-yl)-2-methylpropanamide (66, VU0936154). In a 25 mL round-bottom flask, 2-(4-chlorophenoxy)-2-methylpropanoic acid (29 mg, 1.0 equiv) was dissolved in DMF. HATU (81 mg, 1.6 equiv.) and DIEA (60 uL, 2.5 equiv) was added and stirred for 10 mins. A solution of 4-((5-aminopyrimidin-2-yl)(methyl)amino)benzonitrile (28 mg, 1 equiv) in DMF (0.5 mL) was added to the reaction mixture and allowed to stir overnight. Water was added to it and extracted with ethyl acetate (2 x 15 mL). The combined organic layer was washed with water and concentrated under reduced pressure. Purification of the residue by flash chromatography on silica gel yielded 25 mg (43%) of the title compound. ^{1}H NMR (300 MHz, $CDCl_3$) δ 8.63 (s, 2H), 8.36 (s, 1H), 7.76 – 7.58 (m, 2H), 7.52 – 7.39 (m, 2H), 7.35 – 7.21 (m, 2H), 7.01 – 6.83 (m, 2H), 3.61 (s, 3H), 1.57 (s, 6H); ^{13}C NMR (75 MHz, $CDCl_3$) δ 173.09, 158.35, 152.11, 150.44, 149.22, 132.78, 129.55, 125.03, 124.99, 123.35, 119.04, 107.35, 82.33, 38.10, 24.85. LCMS R_T = 5.88 min; HRMS, calc'd for $C_{22}H_{21}ClN_5O_2^+$ [M+H], 422.1378; found 422.1382.

Characterization of Additional Analogs

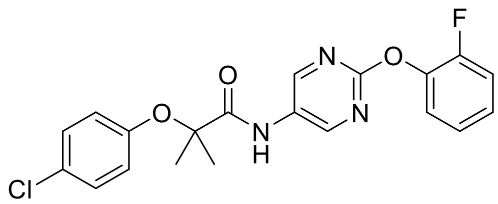

2-(4-Chlorophenoxy)-*N*-(2-(2-fluorophenoxy)pyrimidin-5-yl)-2-methylpropanamide (10). ^{1}H NMR (300 MHz, $CDCl_3$) δ 8.82 (s, 2H), 8.57 (br s, 1H), 7.32 – 7.15 (m, 6H), 6.95 – 6.85 (m, 2H), 1.56 (s, 1H); ^{13}C NMR (75 MHz, $CDCl_3$) δ 173.14, 161.13, 156.24, 152.94, 151.90, 151.36, 140.46, 140.29, 129.73, 129.57, 129.28, 128.81, 126.82, 126.73, 124.77, 124.71, 123.67, 123.57, 121.65, 117.01, 116.77, 82.38, 25.08, 24.78. LCMS R_T = 5.56 min; HRMS, calc'd for $C_{20}H_{18}ClFN_3O_3^+$ [M+H], 402.1015; found 402.102.

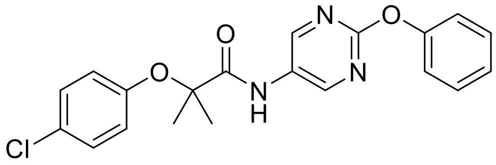

2-(4-Chlorophenoxy)-2-methyl-N-(2-phenoxypyrimidin-5-yl)propenamide (15). ^{1}H NMR (300 MHz, $CDCl_3$) δ 8.80 (s, 2H), 8.60 (br s, 1H), 7.46 – 7.38 (m, 2H), 7.31 – 7.15 (m, 5H), 6.94 – 6.88 (m, 2H), 1.57 (s, 6H); ^{13}C NMR (75 MHz, $CDCl_3$) δ 173.15, 161.99, 153.12, 151.98, 151.49, 129.67, 129.56, 128.45, 125.47, 123.46, 121.47, 82.35, 24.80. LCMS R_T = 5.87 min; HRMS, calc'd for $C_{20}H_{19}ClFN_3O_3^+$ [M+H], 384.1109; found 384.1114.

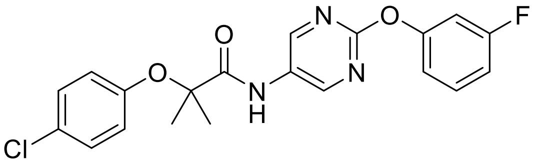

2-(4-Chlorophenoxy)-*N*-(2-(3-fluorophenoxy)pyrimidin-5-yl)-2-methylpropanamide (16). ^{1}H NMR (300 MHz, $CDCl_3$) δ 8.84 (s, 2H), 8.73 (br s, 1H), 7.42 – 7.14 (m, 3H), 7.01 – 6.82 (m, 5H), 1.57 (s, 6H); ^{13}C NMR (75 MHz, $CDCl_3$) δ 173.26, 164.75, 161.47, 161.33, 154.04, 153.90, 151.94, 151.43, 130.48, 130.35, 129.64, 129.55, 128.93, 123.47, 117.18, 117.14, 112.61, 112.34, 109.62, 109.30, 82.32, 24.78. LCMS R_T = 5.88 min; HRMS, calc'd for $C_{20}H_{18}ClFN_3O_3^+$ [M+H], 402.1015; found 402.1018.

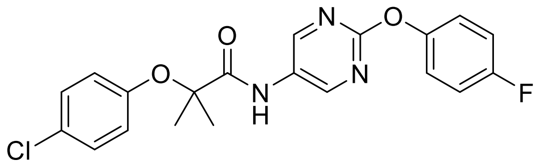

2-(4-Chlorophenoxy)-*N*-(2-(4-fluorophenoxy)pyrimidin-5-yl)-2-methylpropanamide (17). ^{1}H NMR (300 MHz, $CDCl_3$) δ 8.81 (s, 2H), 8.68 (br s, 1H), 7.31 – 7.23 (m, 2H), 7.19 – 7.04 (m, 4H), 6.94 – 6.87 (m, 2H), 1.57 (s, 6H); ^{13}C NMR (75 MHz, $CDCl_3$) δ 173.23, 161.83, 161.59, 158.35, 151.98, 151.48, 148.90, 148.86, 129.63, 129.55, 128.65, 123.43, 123.00, 122.88, 116.46, 116.15, 82.32, 24.79. LCMS R_T = 5.54 min; HRMS, calc'd for $C_{20}H_{18}ClFN_3O_3^+$ [M+H], 402.1015; found 402.1015.

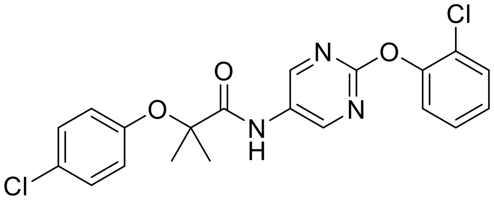

2-(4-Chlorophenoxy)-*N*-(2-(2-chlorophenoxy)pyrimidin-5-yl)-2-methylpropanamide (18). ^{1}H NMR (300 MHz, $CDCl_3$) δ 8.84 (s, 2H), 8.77 (br s), 7.47 (m, 1H), 7.37 – 7.14 (m, 4H), 6.94 – 6.81 (m, 3H), 1.55 (s, 6H); ^{13}C NMR (75 MHz, $CDCl_3$) δ 173.17, 161.17, 151.95, 151.46, 149.06, 130.61, 129.64, 129.55, 129.18, 128.75, 128.06, 127.33, 126.81, 123.75, 123.53, 82.34, 24.79. LCMS R_T = 5.70 min; HRMS, calc'd for $C_{20}H_{18}Cl_2N_3O_3^+$ [M+H], 418.0720; found 418.0726.

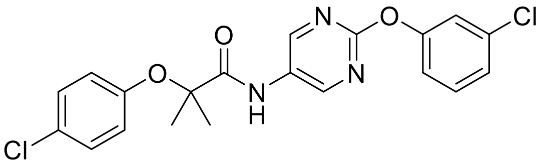

2-(4-Chlorophenoxy)-*N*-(2-(3-chlorophenoxy)pyrimidin-5-yl)-2-methylpropanamide (19). ^{1}H NMR (300 MHz, $CDCl_3$) δ 8.90 (br s, 1H), 8.86 (s, 2H), 7.37 – 7.13 (m, 4H), 7.06 (m,

1H), 6.92 – 6.81 (m, 3H), 1.56 (s, 6H); ^{13}C NMR (75 MHz, $CDCl_3$) δ 173.30, 161.29, 153.56, 151.96, 151.44, 134.83, 130.42, 129.53, 128.99, 125.71, 123.42, 122.08, 119.80, 82.27, 24.79. LCMS R_T = 5.49 min; HRMS, calc'd for $C_{20}H_{18}Cl_2N_3O_3^+$ [M+H], 418.0720; found 418.0721.

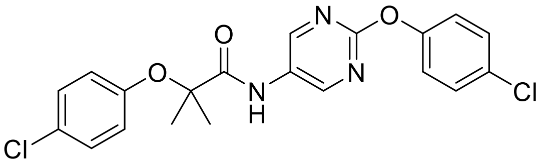

2-(4-Chlorophenoxy)-*N*-(2-(4-chlorophenoxy)pyrimidin-5-yl)-2-methylpropanamide (20). ^{1}H NMR (300 MHz, $CDCl_3$) *δ* 8.83 (s, 2H), 8.70 (br s, 1H), 7.41 – 7.34 (m, 2H), 7.31 – 7.24 (m, 2H), 7.21 – 7.08 (m, 2H), 6.95 – 6.81 (m, 2H), 1.56 (s, 6H); ^{13}C NMR (75 MHz, $CDCl_3$) δ 173.22, 161.59, 151.92, 151.54, 151.44, 130.76, 129.75, 129.67, 129.56, 128.77, 123.49, 122.89, 82.34, 24.79. LCMS R_T = 5.84 min; HRMS, calc'd for $C_{20}H_{18}Cl_2N_3O_3^+$ [M+H], 418.0720; found 418.072.

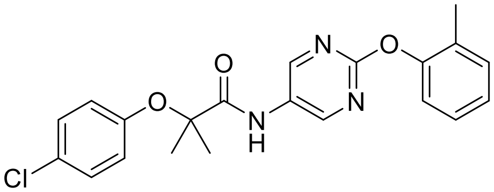

2-(4-Chlorophenoxy)-2-methyl-*N*-(2-(*o*-tolyloxy)pyrimidin-5-yl)propanamide (21). ^{1}H NMR (300 MHz, $CDCl_3$) *δ* 8.80 (s, 2H), 8.52 (br s, 1H), 7.33 – 7.07 (m, 6H), 6.97 – 6.88 (m, 2H), 2.19 (s, 3H), 1.57 (s, 6H); ^{13}C NMR (75 MHz, $CDCl_3$) δ 173.12, 161.90, 151.92, 151.63, 151.47, 131.41, 130.61, 129.74, 129.58, 128.16, 127.16, 125.87, 123.56, 121.82, 82.39, 24.80, 16.32. LCMS R_T = 5.52 min; HRMS, calc'd for $C_{21}H_{21}ClN_3O_3^+$ [M+H], 398.1266; found 398.1266.

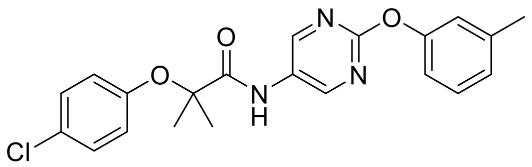

2-(4-Chlorophenoxy)-2-methyl-*N*-(2-(*m*-tolyloxy)pyrimidin-5-yl)propanamide (22). ^{1}H NMR (300 MHz, $CDCl_3$) δ 8.81 (s, 2H), 8.58 (br s, 1H), 7.35 – 7.25 (m, 3H), 7.06 (m, 1H), 7.01 – 6.88 (m, 4H), 2.38 (s, 3H), 1.57 (s, 6H); ^{13}C NMR (75 MHz, $CDCl_3$) δ 173.14, 162.13, 153.05, 151.95, 151.50, 139.91, 129.68, 129.57, 129.40, 128.33, 126.37, 123.51, 122.04, 118.42, 82.36, 24.80, 21.44. LCMS R_T = 5.56 min; HRMS, calc'd for $C_{21}H_{21}ClN_3O_3^+$ [M+H], 398.1266; found 398.127.

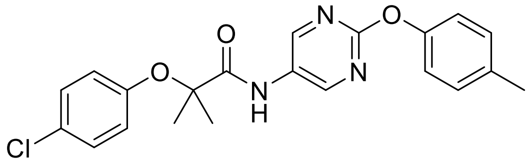

2-(4-Chlorophenoxy)-2-methyl-*N*-(2-(*p*-tolyloxy)pyrimidin-5-yl)propanamide (23). ^{1}H NMR (300 MHz, $CDCl_3$) δ 8.79 (s, 2H), 8.68 (s, 1H), 7.36 – 7.17 (m, 4H), 7.11 – 7.00 (m, 2H), 6.97 – 6.82 (m, 2H), 2.36 (s, 3H), 1.56 (s, 6H); ^{13}C NMR (75 MHz, $CDCl_3$) δ 173.18, 162.18, 152.01, 151.52, 150.80, 135.14, 130.26, 129.56, 129.54, 128.37, 123.43, 121.24, 82.30, 24.81, 20.97. LCMS R_T = 5.69 min; HRMS, calc'd for $C_{21}H_{21}ClN_3O_3^+$ [M+H], 398.1266; found 398.1271.

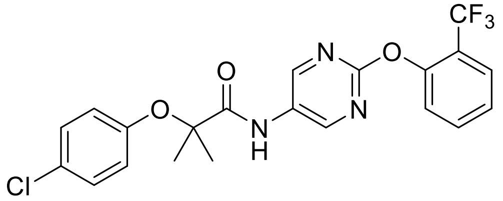

2-(4-Chlorophenoxy)-2-methyl-*N*-(2-(2-(trifluoromethyl)phenoxy)pyrimidin-5-yl)propanamide (24). ^{1}H NMR (300 MHz, $CDCl_3$) δ 8.83 (s, 2H), 8.57 (bs, 1H), 7.73 (dd, *J* = 7.9, 1.9 Hz, 1H), 7.62 (td, *J* = 8.2, 2.0 Hz, 1H), 7.37 (tt, *J* = 7.6, 0.9 Hz, 1H), 7.32 – 7.21 (m,

3H), 7.03 – 6.83 (m, 2H), 1.57 (s, 6H); ^{13}C NMR (75 MHz, $CDCl_3$) δ 173.14, 161.57, 151.87, 151.34, 150.56, 133.21, 129.80, 129.59, 128.89, 127.25, 127.19, 125.60, 124.13, 123.62, 121.24, 82.41, 24.78. LCMS R_T = 5.67 min; HRMS, calc'd for $C_{21}H_{18}ClF_3N_3O_3^+$ [M+H], 452.0983; found 452.0985.

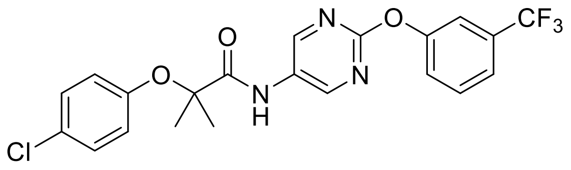

2-(4-Chlorophenoxy)-2-methyl-*N*-(2-(3-(trifluoromethyl)phenoxy)pyrimidin-5-yl)propanamide (25). ^{1}H NMR (300 MHz, $CDCl_3$) *δ* 8.85 (s, 2H), 8.61 (bs, 1H), 7.59 – 7.46 (m, 3H), 7.42 – 7.36 (m, 1H), 7.33 – 7.25 (m, 2H), 7.00 – 6.87 (m, 2H), 1.57 (s, 6H); ^{13}C NMR (75 MHz, $CDCl_3$) δ 173.21, 161.33, 153.20, 151.86, 151.40, 132.35, 131.92, 130.23, 129.81, 129.60, 129.27, 128.97, 125.00, 124.98, 123.59, 122.21, 122.16, 118.77, 118.72, 82.41, 24.78. LCMS R_T = 5.78 min; HRMS, calc'd for $C_{21}H_{18}ClF_3N_3O_3^+$ [M+H], 452.0983; found 452.0989.

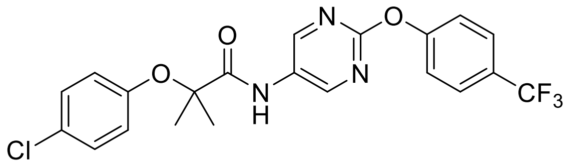

2-(4-Chlorophenoxy)-2-methyl-*N*-(2-(4-(trifluoromethyl)phenoxy)pyrimidin-5-yl)propanamide (26). ^{1}H NMR (300 MHz, $CDCl_3$) *δ* 8.85 (s, 2H), 8.69 (s, 1H), 7.69 (dd, *J* = 9.1, 0.6 Hz, 2H), 7.46 – 7.20 (m, 4H), 7.09 – 6.72 (m, 2H), 1.57 (s, 6H); ^{13}C NMR (75 MHz, $CDCl_3$) δ 173.26, 161.16, 155.62, 151.88, 151.40, 150.32, 129.75, 129.62, 129.58, 129.09, 127.09, 127.04, 123.54, 121.72, 82.37, 24.78. LCMS R_T = 5.94 min; HRMS, calc'd for $C_{21}H_{18}ClF_3N_3O_3^+$ [M+H], 452.0983; found 452.0987.

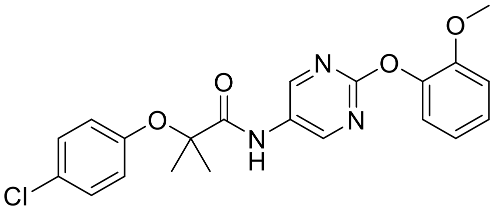

2-(4-Chlorophenoxy)-*N*-(2-(2-methoxyphenoxy)pyrimidin-5-yl)-2-methylpropanamide (27). ^{1}H NMR (300 MHz, $CDCl_3$) δ 8.78 (s, 3H), 7.33 – 7.12 (m, 4H), 7.04 – 6.95 (m, 2H), 6.93 – 6.81 (m, 2H), 3.71 (s, 3H), 1.55 (s, 6H); ^{13}C NMR (75 MHz, $CDCl_3$) δ 173.22, 161.75, 152.07, 151.52, 151.45, 141.87, 129.49, 129.44, 129.13, 128.39, 126.63, 123.42, 122.74, 121.01, 112.77, 82.25, 55.83, 24.80. LCMS R_T = 5.44 min; HRMS, calc'd for $C_{21}H_{21}ClN_3O_4^+$ [M+H], 414.1215; found 414.1216.

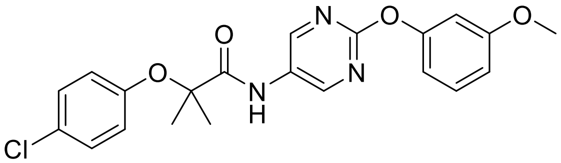

2-(4-Chlorophenoxy)-*N*-(2-(3-methoxyphenoxy)pyrimidin-5-yl)-2-methylpropanamide (28). ^{1}H NMR (300 MHz, $CDCl_3$) δ 1H NMR (300 MHz, CDCl3) δ 8.83 (s, 2H), 8.64 (s, 1H), 7.47 – 7.20 (m, 3H), 7.04 – 6.87 (m, 2H), 6.87 – 6.63 (m, 3H), 3.80 (s, 3H), 1.56 (s, 6H); ^{13}C NMR (75 MHz, $CDCl_3$) δ 173.21, 161.84, 160.74, 154.06, 151.94, 151.51, 130.08, 129.67, 129.56, 129.21, 128.52, 123.52, 113.62, 111.26, 107.53, 82.35, 55.42, 24.79. LCMS R_T = 5.52 min; HRMS, calc'd for $C_{21}H_{21}ClN_3O_4^+$ [M+H], 414.1215; found 414.1217.

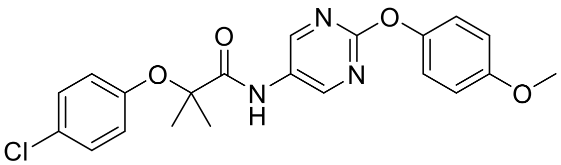

2-(4-Chlorophenoxy)-*N*-(2-(4-methoxyphenoxy)pyrimidin-5-yl)-2-methylpropanamide (29). ^{1}H NMR (300 MHz, $CDCl_3$) δ ^{1}H NMR (300 MHz, CDCl3) δ 8.89 (s, 1H), 8.79 (s, 2H), 7.30 – 7.19 (m, 2H), 7.12 – 7.01 (m, 2H), 6.96 – 6.79 (m, 4H), 3.79 (s, 3H), 1.56 (s,5H); ^{13}C

NMR (75 MHz, $CDCl_3$) δ 173.26, 162.18, 156.95, 152.11, 151.55, 146.48, 129.48, 129.33, 128.49, 123.27, 122.37, 114.69, 82.17, 55.55, 24.81. LCMS R_T = 5.44 min; HRMS, calc'd for $C_{21}H_{21}ClN_3O_4^+$ [M+H], 414.1215; found 414.1219.

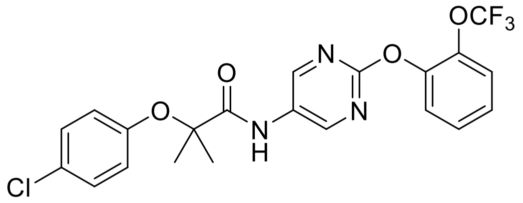

2-(4-Chlorophenoxy)-2-methyl-*N*-(2-(2-(trifluoromethoxy)phenoxy)pyrimidin-5-yl)propanamide (30). ^1H NMR (300 MHz, $CDCl_3$) *δ* ^1H NMR (300 MHz, CDCl3) δ 8.83 (s, 2H), 8.57 (s, 1H), 7.42 – 7.35 (m, 2H), 7.33 – 7.27 (m, 4H), 6.97 – 6.85 (m, 2H), 1.57 (s, 6H); ^{13}C NMR (75 MHz, $CDCl_3$) δ 173.11, 161.22, 151.87, 151.30, 144.86, 129.80, 129.59, 128.82, 128.77, 127.83, 126.54, 124.05, 123.65, 122.42, 120.23, 82.43, 24.78. LCMS R_T = 5.73 min; HRMS, calc'd for $C_{21}H_{18}ClF_3N_3O_4^+$ [M+H], 468.0932; found 468.0937.

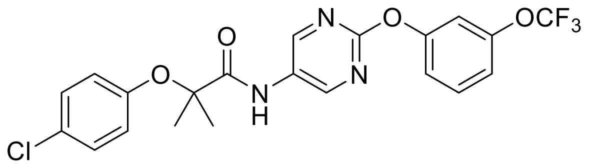

2-(4-Chlorophenoxy)-2-methyl-*N*-(2-(3-(trifluoromethoxy)phenoxy)pyrimidin-5-yl)propanamide (31). ^1H NMR (300 MHz, $CDCl_3$) *δ* 8.85 (s, 2H), 8.61 (s, 1H), 7.50 – 7.41 (m, 1H), 7.34 – 7.25 (m, 2H), 7.20 – 7.08 (m, 3H), 7.01 – 6.82 (m, 2H), 1.57 (s, 6H); ^{13}C NMR (75 MHz, $CDCl_3$) δ 173.20, 161.31, 153.82, 151.87, 151.38, 149.81, 149.79, 130.36, 129.79, 129.60, 128.94, 123.58, 119.83, 117.63, 114.70, 82.40, 24.78. LCMS R_T = 5.84 min; HRMS, calc'd for $C_{21}H_{18}ClF_3N_3O_4^+$ [M+H], 468.0932; found 468.0937.

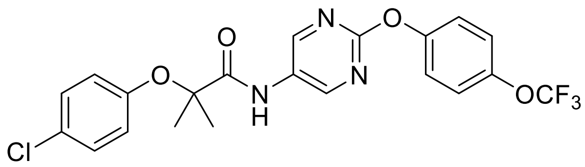

2-(4-Chlorophenoxy)-2-methyl-*N*-(2-(4-(trifluoromethoxy)phenoxy)pyrimidin-5-yl)propanamide (32). ^{1}H NMR (300 MHz, $CDCl_3$) δ 8.84 (s, 2H), 8.61 (s, 1H), 7.52 – 7.37 (m, 1H), 7.33 – 7.27 (m, 2H), 7.18 – 7.08 (m, 3H), 6.97 – 6.88 (m, 2H), 1.57 (s, 3H); ^{13}C NMR (75 MHz, $CDCl_3$) δ 173.20, 153.83, 151.88, 151.39, 149.79, 130.36, 129.79, 129.60, 128.95, 123.58, 119.83, 117.63, 114.69, 82.40, 24.78. LCMS R_T = 6.00 min; HRMS, calc'd for $C_{21}H_{18}ClF_3N_3O_4^+$ [M+H], 468.0932; found 468.0936.

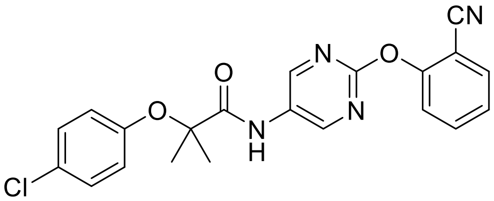

2-(4-Chlorophenoxy)-*N*-(2-(2-cyanophenoxy)pyrimidin-5-yl)-2-methylpropanamide (33). ^{1}H NMR (300 MHz, $CDCl_3$) δ 8.87 (s, 2H), 8.66 (s, 1H), 7.93 – 7.59 (m, 2H), 7.37 (dd, J = 7.7, 1.1 Hz, 3H), 7.28 – 7.22 (m, 1H), 7.07 – 6.83 (m, 2H), 1.57 (s, 1H); ^{13}C NMR (75 MHz, $CDCl_3$) δ 173.19, 160.83, 154.74, 151.87, 151.26, 134.42, 133.66, 129.78, 129.59, 129.46, 125.89, 123.66, 122.90, 115.40, 107.35, 82.40, 24.78. LCMS R_T = 5.30 min; HRMS, calc'd for $C_{21}H_{18}ClN_4O_3^+$ [M+H], 409.1062; found 409.1073.

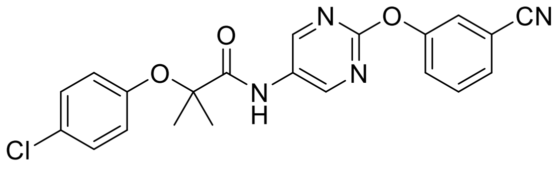

2-(4-Chlorophenoxy)-*N*-(2-(3-cyanophenoxy)pyrimidin-5-yl)-2-methylpropanamide (34). ^{1}H NMR (300 MHz, $CDCl_3$) δ 8.86 (s, 2H), 8.67 (s, 1H), 7.64 – 7.49 (m,3H), 7.49 – 7.39 (m, 1H), 7.34 – 7.20 (m, 2H), 7.02 – 6.81 (m, 2H), 1.58 (s, 6H); ^{13}C NMR (75 MHz, $CDCl_3$) δ

173.25, 160.96, 153.25, 151.87, 151.36, 130.62, 129.78, 129.60, 129.28, 129.03, 126.43, 125.21, 123.58, 118.05, 113.55, 82.39, 24.78. LCMS R_T = 5.28 min; HRMS, calc'd for $C_{21}H_{18}ClN_4O_3^+$ [M+H], 409.1062; found 409.1066.

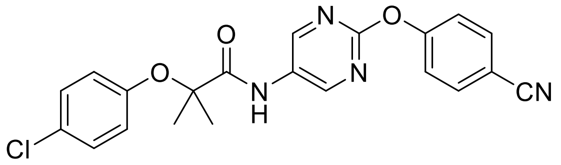

2-(4-Chlorophenoxy)-*N*-(2-(4-cyanophenoxy)pyrimidin-5-yl)-2-methylpropanamide (35). ^{1}H NMR (300 MHz, $CDCl_3$) δ 8.87 (s, 2H), 8.64 (s, 1H), 7.86 – 7.62 (m, 2H), 7.34 – 7.24 (m, 4H), 6.93 (d, *J* = 9.0 Hz, 2H), 1.58 (s, 6H); ^{13}C NMR (75 MHz, $CDCl_3$) δ 173.26, 160.77, 156.51, 151.82, 151.34, 133.95, 129.86, 129.62, 129.37, 123.62, 122.26, 118.46, 109.01, 82.41, 24.78. LCMS R_T = 5.42 min; HRMS, calc'd for $C_{21}H_{18}ClN_4O_3^+$ [M+H], 409.1062; found 409.1069.

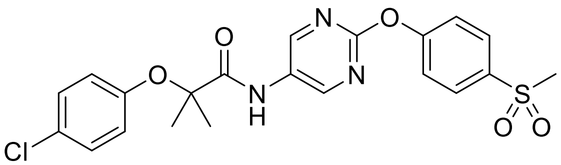

2-(4-Chlorophenoxy)-2-methyl-*N*-(2-(4-(methylsulfonyl)phenoxy)pyrimidin-5-yl)propanamide (36). ^{1}H NMR (300 MHz, $CDCl_3$) δ 9.65 (s, 1H), 8.92 (s, 2H), 8.00 (dd, *J* = 8.8, 1.1 Hz, 2H), 7.43-7.76 (m, 2H), 7.36 – 7.12 (m, 2H), 6.94-6.88 (m, 2H), 3.12 (s, 3H), 1.60 (s, 6H); ^{13}C NMR (75 MHz, $CDCl_3$) δ 174.13, 160.32, 157.39, 152.47, 151.74, 136.68, 130.08, 129.40, 128.91, 122.60, 122.05, 115.97, 81.71, 44.46, 24.69. LCMS R_T = 5.06 min; HRMS, calc'd for $C_{21}H_{21}ClN_4O_5S^+$ [M+H], 462.0885; found 462.0888.

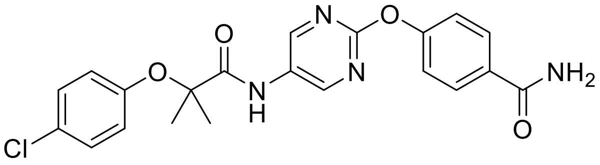

4-((5-(2-(4-chlorophenoxy)-2-methylpropanamido)pyrimidin-2-yl)oxy)benzamide (37). ^{1}H NMR (300 MHz, $CDCl_3$) δ 8.86 (s, 2H), 8.66 (s, 1H), 8.05 – 7.70 (m, 2H), 7.32 – 7.27 (m, 3H), 7.26 (d, *J* = 3.3 Hz, 1H), 7.00 – 6.83 (m, 2H), 5.93 (d, *J* = 91.3 Hz, 2H), 1.58 (s, 6H); ^{13}C NMR (75 MHz, $CDCl_3$) δ 173.24, 168.47, 161.32, 156.08, 151.90, 151.44, 130.29, 129.77, 129.60, 129.20, 129.00, 123.55, 121.51, 82.40, 24.79. LCMS R_T = 4.56 min; HRMS, calc'd for $C_{21}H_{20}ClN_4O_4^+$ [M+H], 427.1168; found 427.1172.

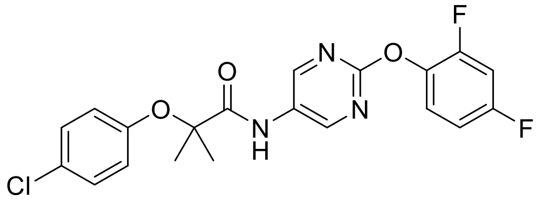

2-(4-chlorophenoxy)-*N*-(2-(2,4-difluorophenoxy)pyrimidin-5-yl)-2-methylpropanamide (38). ^{1}H NMR (300 MHz, $CDCl_3$) δ 8.82 (s, 2H), 8.65 (s, 1H), 7.33 – 7.19 (m, 3H), 7.01 – 6.75 (m, 4H), 1.56 (s, 6H); ^{13}C NMR (75 MHz, $CDCl_3$) δ 173.18, 160.98, 158.21, 156.14, 152.96, 152.79, 151.91, 151.36, 136.84, 136.78, 136.67, 136.62, 129.71, 129.57, 128.99, 124.22, 124.19, 124.09, 124.06, 123.55, 111.61, 111.56, 111.31, 111.25, 105.65, 105.35, 105.29, 105.00, 82.36, 24.78. LCMS R_T = 5.68 min; HRMS, calc'd for $C_{20}H_{17}ClF_2N_3O_3^+$ [M+H], 420.0921; found 420.0924.

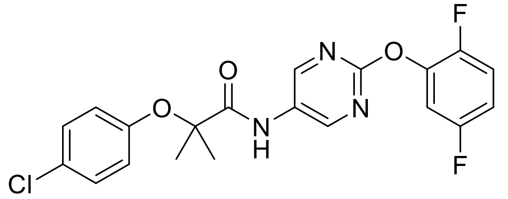

2-(4-chlorophenoxy)-*N*-(2-(2,5-difluorophenoxy)pyrimidin-5-yl)-2-methylpropanamide (39). ^{1}H NMR (300 MHz, $CDCl_3$) δ 8.84 (s, 2H), 8.67 (s, 1H), 7.35 – 7.22 (m, 2H), 7.15 (td, *J* = 9.4, 5.0 Hz, 1H), 7.02 (ddd, *J* = 8.3, 6.1, 3.1 Hz, 1H), 6.98 – 6.82 (m, 3H), 1.56 (s, 6H); ^{13}C NMR (75 MHz, $CDCl_3$) δ 13C NMR (75 MHz, CDCl3) δ 173.19, 171.24, 160.65, 160.03, 160.00, 156.80, 156.76, 152.69, 152.65, 151.90, 151.31, 149.44, 149.40,

140.88, 140.69, 140.54, 129.72, 129.64, 129.57, 129.20, 123.55, 117.31, 117.18, 117.03, 116.90, 113.19, 113.09, 112.87, 112.78, 111.50, 111.48, 111.16, 111.14, 82.36, 24.77. LCMS R_T = 5.77 min; HRMS, calc'd for $C_{20}H_{17}ClF_2N_3O_3^+$ [M+H], 420.0921; found 420.0919.

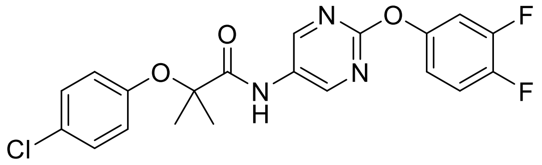

2-(4-chlorophenoxy)-*N*-(2-(3,4-difluorophenoxy)pyrimidin-5-yl)-2-methylpropanamide (41). ^{1}H NMR (300 MHz, $CDCl_3$) δ 8.84 (s, 2H), 8.68 (s, 1H), 7.34 – 7.25 (m, 2H), 7.20 (dt, *J* = 10.0, 8.9 Hz, 1H), 7.06 (ddd, *J* = 10.7, 6.7, 2.8 Hz, 1H), 6.98 – 6.86 (m, 3H), 1.57 (s, 6H); ^{13}C NMR (75 MHz, $CDCl_3$) δ 173.24, 161.38, 152.03, 151.89, 151.84, 151.40, 149.74, 149.57, 148.76, 148.72, 148.65, 148.61, 148.53, 146.48, 146.32, 129.73, 129.63, 129.58, 128.98, 123.52, 117.62, 117.60, 117.56, 117.51, 117.48, 117.42, 117.37, 117.35, 111.69, 111.43, 82.36, 24.78. LCMS R_T = 5.77 min; HRMS, calc'd for $C_{20}H_{17}ClF_2N_3O_3^+$ [M+H], 420.0921; found 420.0921.

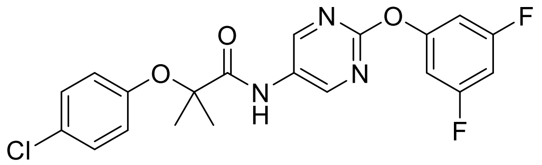

2-(4-chlorophenoxy)-*N*-(2-(3,5-difluorophenoxy)pyrimidin-5-yl)-2-methylpropanamide (42). ^{1}H NMR (300 MHz, $CDCl_3$) δ 8.86 (s, 2H), 8.69 (s, 1H), 7.38 – 7.21 (m, 2H), 6.99 – 6.89 (m, 2H), 6.85 – 6.65 (m, 3H), 1.57 (s, 6H); ^{13}C NMR (75 MHz, $CDCl_3$) δ 173.22, 164.97, 164.78, 161.68, 161.48, 160.87, 154.54, 151.84, 151.34, 129.81, 129.60, 129.24, 123.59, 105.65, 105.52, 105.39, 105.30, 105.27, 101.50, 101.16, 100.82, 82.40, 24.77. LCMS R_T = 5.78 min; HRMS, calc'd for $C_{20}H_{17}ClF_2N_3O_3^+$ [M+H], 420.0921; found 420.0923.

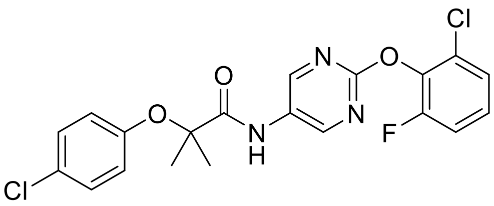

***N*-(2-(2-Chloro-6-fluorophenoxy)pyrimidin-5-yl)-2-(4-chlorophenoxy)-2-methylpropanamide (43).** ^{1}H NMR (300 MHz, $CDCl_3$) δ 8.83 (s, 2H), 8.70 (s, 1H), 7.34 – 7.23 (m, 3H), 7.23 – 7.05 (m, 2H), 6.95 – 6.85 (m, 2H), 1.56 (s, 6H); ^{13}C NMR (75 MHz, $CDCl_3$) δ 13C NMR (75 MHz, CDCl3) δ 173.18, 160.28, 157.39, 154.06, 151.93, 151.40, 137.80, 137.61, 129.67, 129.59, 129.55, 129.25, 129.08, 129.05, 126.55, 126.44, 125.63, 125.58, 123.57, 115.43, 115.18, 82.34, 24.78. LCMS R_T = 5.82 min; HRMS, calc'd for $C_{20}H_{17}Cl_2FN_3O_3^+$ [M+H], 436.0626; found 436.0627.

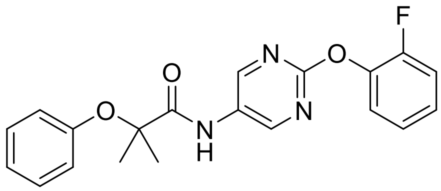

***N*-(2-(2-Fluorophenoxy)pyrimidin-5-yl)-2-methyl-2-phenoxypropanamide (44).** ^{1}H NMR (300 MHz, $CDCl_3$) δ 8.83 (s, 2H), 8.60 (s, 1H), 7.38 – 7.27 (m, 3H), 7.25 – 7.10 (m, 4H), 7.02 – 6.91 (m, 2H), 1.58 (s, 6H); ^{13}C NMR (75 MHz, $CDCl_3$) δ 173.56, 161.15, 153.38, 152.97, 151.32, 140.36, 130.53, 129.56, 128.88, 126.78, 126.69, 124.76, 124.70, 124.37, 123.68, 122.19, 121.00, 117.01, 116.77, 81.97, 24.90. LCMS R_T = 5.29 min; HRMS, calc'd for $C_{20}H_{19}FN_3O_3^+$ [M+H], 368.1405; found 368.1408.

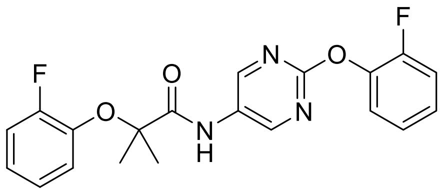

2-(2-Fluorophenoxy)-*N*-(2-(2-fluorophenoxy)pyrimidin-5-yl)-2-methylpropanamide (45). ^1H NMR (300 MHz, $CDCl_3$) δ 8.96 (s, 1H), 8.86 (s, 2H), 7.53 – 6.92 (m, 8H), 1.58 (s, 6H); ^{13}C NMR (75 MHz, $CDCl_3$) δ 13C NMR (75 MHz, CDCl3) δ 173.03, 161.07, 157.83, 156.28, 154.58, 152.97, 151.27, 140.95, 140.79, 140.53, 140.37, 129.03, 126.76, 126.66, 125.87, 125.77, 125.22, 124.75, 124.70, 124.60, 124.55, 123.69, 117.00, 116.75, 83.97, 24.68, 24.66. LCMS R_T = 5.33 min; HRMS, calc'd for $C_{20}H_{18}F_2N_3O_3^+$ [M+H], 386.1311; found 386.1314.

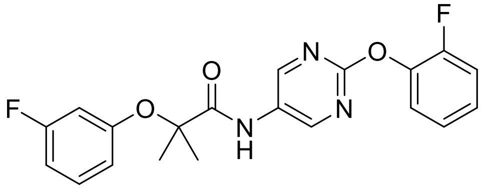

2-(3-Fluorophenoxy)-*N*-(2-(2-fluorophenoxy)pyrimidin-5-yl)-2-methylpropanamide (46). ^1H NMR (300 MHz, $CDCl_3$) δ 8.81 (s, 2H), 8.59 (s, 1H), 7.38 – 7.10 (m, 5H), 6.85 (tdd, *J* = 8.3, 2.5, 0.9 Hz, 1H), 6.78 – 6.67 (m, 2H), 1.59 (s, 6H); ^{13}C NMR (75 MHz, $CDCl_3$) δ 173.10, 164.75, 161.47, 161.16, 156.25, 154.78, 154.64, 152.94, 151.43, 140.47, 140.31, 130.34, 130.22, 128.81, 126.81, 126.71, 124.76, 124.71, 123.68, 117.47, 117.42, 117.00, 116.76, 111.45, 111.17, 109.95, 109.64, 82.45, 24.85. LCMS R_T = 5.21 min; HRMS, calc'd for $C_{20}H_{18}F_2N_3O_3^+$ [M+H], 386.1311; found 386.1314.

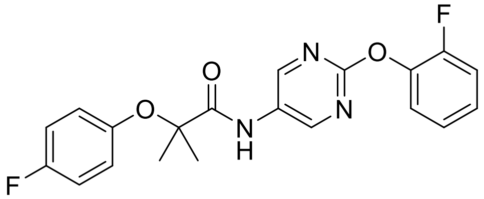

2-(4-Fluorophenoxy)-*N*-(2-(2-fluorophenoxy)pyrimidin-5-yl)-2-methylpropanamide (47). ^1H NMR (300 MHz, $CDCl_3$) δ 8.83 (s, 2H), 8.62 (s, 1H), 7.32 – 7.26 (m, 1H), 7.24 – 7.15 (m, 3H), 7.08 – 6.90 (m, 4H), 1.55 (s, 6H); ^{13}C NMR (75 MHz, $CDCl_3$) δ 173.22, 161.17, 157.92, 156.28, 152.97, 151.30, 149.13, 149.09, 140.53, 140.37, 128.84, 126.77, 126.67, 124.74,

124.69, 124.09, 123.98, 123.68, 117.00, 116.76, 116.26, 115.95, 82.39, 24.73. LCMS R_T = 5.24 min; HRMS, calc'd for $C_{20}H_{18}F_2N_3O_3^+$ [M+H], 386.1311; found 386.1347.

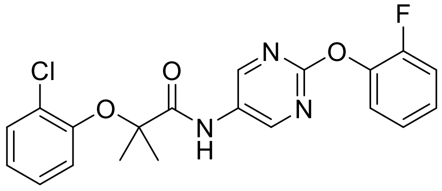

2-(2-Chlorophenoxy)-*N*-(2-(2-fluorophenoxy)pyrimidin-5-yl)-2-methylpropanamide (48). ^{1}H NMR (300 MHz, $CDCl_3$) δ 9.26 (s, 1H), 8.88 (s, 2H), 7.44 (dd, *J* = 7.9, 1.7 Hz, 1H), 7.32 – 7.00 (m, 7H), 1.64 (s, 6H); δ ^{13}C NMR (75 MHz, CDCl3) δ 172.98, 161.09, 156.29, 152.98, 151.08, 149.89, 140.53, 140.37, 130.68, 129.13, 127.92, 127.76, 126.77, 126.67, 125.21, 124.75, 124.70, 123.71, 123.69, 122.70, 117.00, 116.75, 84.23, 24.83. LCMS R_T = 5.55 min; HRMS, calc'd for $C_{20}H_{18}ClFN_3O_3^+$ [M+H], 402.1015; found 402.1024.

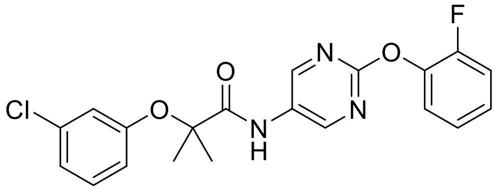

2-(3-Chlorophenoxy)-*N*-(2-(2-fluorophenoxy)pyrimidin-5-yl)-2-methylpropanamide (49). ^{1}H NMR (300 MHz, $CDCl_3$) δ 8.82 (s, 2H), 8.55 (s, 1H), 7.35 – 7.07 (m, 6H), 7.01 (t, *J* = 2.2 Hz, 1H), 6.87 (ddd, *J* = 8.2, 2.3, 1.1 Hz,1H), 1.59 (s, 6H); ^{13}C NMR (75 MHz, $CDCl_3$) δ 173.03, 161.18, 156.25, 154.17, 152.95, 151.43, 140.47, 140.31, 134.87, 130.27, 126.82, 126.72, 124.77, 124.72, 124.65, 123.67, 122.79, 120.14, 117.01, 116.77, 82.56, 24.86. LCMS R_T = 5.6 min; HRMS, calc'd for $C_{20}H_{18}ClFN_3O_3^+$ [M+H], 402.1015; found 402.1014.

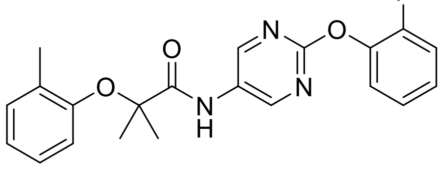

***N*-(2-(2-Fluorophenoxy)pyrimidin-5-yl)-2-methyl-2-(*o*-tolyloxy)propanamide (50).** ^{1}H NMR (300 MHz, $CDCl_3$) δ 8.82 (s, 2H), 8.62 (s, 1H), 7.25 – 7.19 (m, 4H), 7.12 (dddd, *J* = 8.0, 7.4, 1.9, 0.6 Hz, 2H), 7.01 (td, *J* = 7.4, 1.4 Hz, 1H), 6.85 (dd, *J* = 8.1, 1.4 Hz, 1H), 2.29 (s, 3H), 1.61 (s, 6H); ^{13}C NMR (75 MHz, $CDCl_3$) δ 173.95, 161.18, 152.35, 151.28, 131.60, 130.65, 128.88, 126.78, 126.70, 124.76, 124.71, 123.69, 119.68, 117.01, 116.77, 82.03, 25.15, 17.14. LCMS R_T = 5.41 min; HRMS, calc'd for $C_{21}H_{21}FN_3O_3^+$ [M+H], 382.1561; found 382.1567.

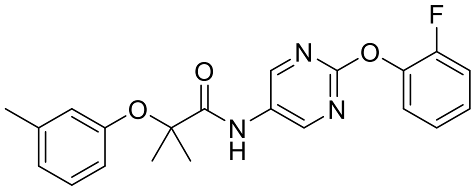

***N*-(2-(2-Fluorophenoxy)pyrimidin-5-yl)-2-methyl-2-(*m*-tolyloxy)propanamide (51).** ^{1}H NMR (300 MHz, $CDCl_3$) δ 8.81 (s, 2H), 8.67 (s, 1H), 7.40 – 7.15 (m, 5H), 6.97-6.93 (m, 1H), 6.84 – 6.62 (m, 2H), 2.33 (s, 3H), 1.57 (s, 6H); ^{13}C NMR (75 MHz, $CDCl_3$) δ 173.72, 161.10, 156.27, 153.39, 151.34, 140.52, 140.36, 139.71, 129.18, 128.95, 126.77, 126.67, 125.09, 124.75, 124.70, 123.69, 122.88, 118.93, 117.00, 116.76, 81.79, 24.94, 21.43. LCMS R_T = 5.38 min; HRMS, calc'd for $C_{21}H_{21}FN_3O_3^+$ [M+H], 382.1561; found 382.1567.

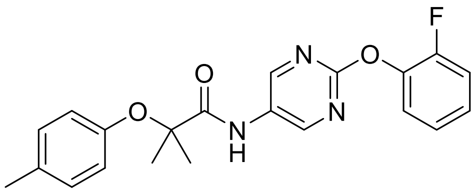

***N*-(2-(2-Fluorophenoxy)pyrimidin-5-yl)-2-methyl-2-(*p*-tolyloxy)propanamide (52).** ^{1}H NMR (300 MHz, $CDCl_3$) δ 8.82 (s, 2H), 8.72 (s, 1H), 7.31 – 7.15 (m, 4H), 7.14 – 7.02 (m, 2H), 6.92 – 6.80 (m, 2H), 2.32 (s, 3H), 1.55 (s, 6H); ^{13}C NMR (75 MHz, $CDCl_3$) δ 173.73, 161.07, 156.27, 152.97, 151.31, 150.99, 140.52, 140.36, 133.98, 130.00, 128.98, 126.77, 126.67, 124.75, 124.70, 123.70, 123.69, 122.15, 117.00, 116.76, 81.82, 24.84, 20.72. LCMS R_T = 5.55 min; HRMS, calc'd for $C_{21}H_{21}FN_3O_3^+$ [M+H], 382.1561; found 382.1568.

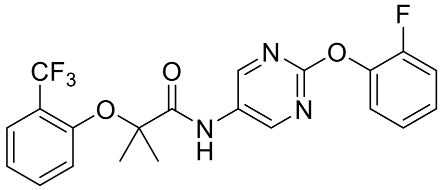

N-(2-(2-Fluorophenoxy)pyrimidin-5-yl)-2-methyl-2-(2-(trifluoromethyl)phenoxy)propanamide (53). ^{1}H NMR (300 MHz, $CDCl_3$) δ 8.85 (s, 2H), 8.81 (s, 1H), 7.75 – 7.59 (m, 1H), 7.51 (tdd, J = 7.6, 1.8, 0.6 Hz, 1H), 7.35 – 6.92 (m, 6H), 1.72 (s, 6H); ^{13}C NMR (75 MHz, $CDCl_3$) δ 172.82, 161.15, 156.28, 152.98, 152.50, 152.48, 151.12, 140.49, 140.33, 133.29, 128.97, 127.73, 127.66, 127.59, 127.52, 126.80, 126.70, 125.59, 124.75, 124.70, 123.69, 122.93, 122.40, 122.01, 121.98, 119.69, 116.99, 116.75, 83.63, 24.84. LCMS R_T = 5.68 min; HRMS, calc'd for $C_{21}H_{18}F_4N_3O_3^+$ [M+H], 436.1279; found 436.1283.

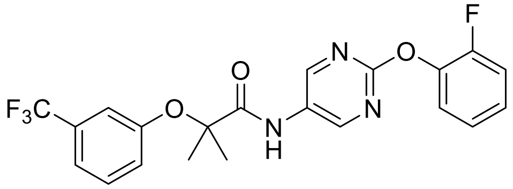

N-(2-(2-Fluorophenoxy)pyrimidin-5-yl)-2-methyl-2-(3-(trifluoromethyl)phenoxy)propanamide (54). ^{1}H NMR (300 MHz, $CDCl_3$) δ 8.82 (s, 2H), 8.50 (s, 1H), 7.55 – 7.40 (m, 2H), 7.31 – 7.25 (m, 1H), 7.25 – 7.11 (m, 5H), 1.61 (s, 6H); ^{13}C NMR (75 MHz, $CDCl_3$) δ 172.82, 161.26, 156.26, 153.65, 152.95, 151.49, 140.48, 140.31, 132.43, 132.00, 130.16, 128.66, 126.83, 126.73, 125.16, 124.77, 124.72, 123.66, 121.14, 121.09, 119.48, 117.01, 116.77, 82.71, 24.82. LCMS R_T = 5.51 min; HRMS, calc'd for $C_{21}H_{18}F_4N_3O_3^+$ [M+H], 436.1279; found 436.1285.

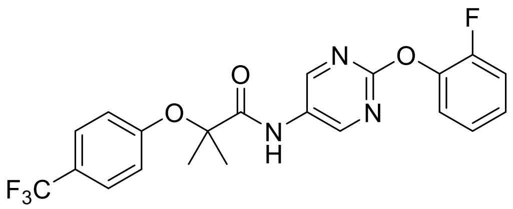

***N*-(2-(2-Fluorophenoxy)pyrimidin-5-yl)-2-methyl-2-(4-(trifluoromethyl)phenoxy)propanamide (55).** ^{1}H NMR (300 MHz, $CDCl_3$) *δ* 8.79 (s, 2H), 8.45 (s, 1H), 7.59 (dd, *J* = 9.1, 2H), 7.32 – 7.15 (m, 4H), 7.06 (dd, *J* = 9.1, Hz, 2H), 1.63 (s, 6H); ^{13}C NMR (75 MHz, $CDCl_3$) δ 172.85, 161.25, 156.36, 156.24, 152.93, 151.46, 140.44, 140.28, 128.67, 127.06, 127.01, 126.96, 126.91, 126.85, 126.75, 124.78, 124.73, 123.65, 121.46, 117.01, 116.77, 82.47, 24.89. LCMS R_T = 5.64 min; HRMS, calc'd for $C_{21}H_{18}F_4N_3O_3^+$ [M+H], 436.1279; found 436.1284.

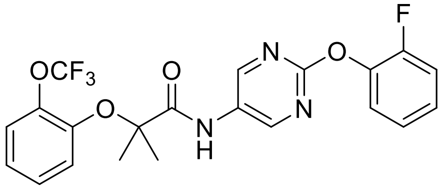

***N*-(2-(2-Fluorophenoxy)pyrimidin-5-yl)-2-methyl-2-(2-(trifluoromethoxy)phenoxy)propanamide (56).** ^{1}H NMR (300 MHz, $CDCl_3$) *δ* 9.50 – 8.38 (m, 3H), 7.47 – 6.82 (m, 8H), 1.62 (s, 6H); ^{13}C NMR (75 MHz, CDCl3) δ 172.83, 161.13, 156.28, 152.98, 151.12, 145.85, 142.09, 140.52, 140.35, 127.84, 126.78, 126.69, 125.04, 124.75, 124.69, 123.70, 123.47, 122.88, 122.34, 118.92, 117.00, 116.76, 84.04, 24.67. LCMS R_T = 5.60 min; HRMS, calc'd for $C_{21}H_{18}F_4N_3O_4^+$ [M+H], 452.1228; found 452.1229.

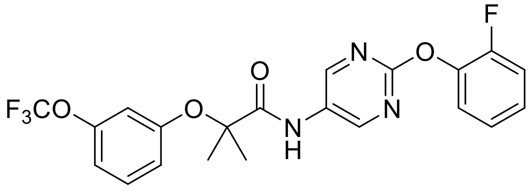

***N*-(2-(2-Fluorophenoxy)pyrimidin-5-yl)-2-methyl-2-(3-(trifluoromethoxy)phenoxy)propanamide (57).** ^{1}H NMR (300 MHz, $CDCl_3$) δ 8.80 (s, 2H), 8.60 (s, 1H), 7.40 – 7.11 (m, 5H), 7.01 (ddt, *J* = 8.3, 2.2, 1.1 Hz, 1H), 6.94 – 6.81 (m, 2H), 1.60 (s, 6H); ^{13}C NMR (75 MHz, CDCl3) δ 172.95, 161.18, 156.24, 154.45, 152.93, 151.49, 149.78, 149.76, 140.45, 140.29, 130.28, 128.77, 126.82, 126.72, 124.76, 124.71, 123.66, 120.04, 117.00, 116.75, 116.60, 115.22, 82.60, 24.78. LCMS R_T = 5.73 min; HRMS, calc'd for $C_{21}H_{18}F_4N_3O_4^+$ [M+H], 452.1228; found 452.1233.

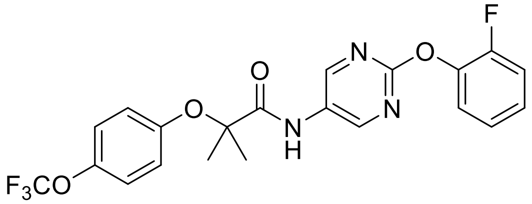

***N*-(2-(2-Fluorophenoxy)pyrimidin-5-yl)-2-methyl-2-(4-(trifluoromethoxy)phenoxy)propanamide (58).** ^{1}H NMR (300 MHz, $CDCl_3$) δ 8.82 (s, 2H), 8.63 (s, 1H), 7.31 – 7.11 (m, 6H), 7.05 – 6.93 (m, 2H), 1.58 (s, 6H); ^{13}C NMR (75 MHz, $CDCl_3$) δ 173.07, 161.15, 156.24, 152.94, 151.78, 151.36, 145.47, 145.45, 140.46, 140.30, 128.83, 126.82, 126.73, 124.77, 124.72, 123.66, 123.35, 122.25, 122.13, 118.73, 117.00, 116.76, 82.46, 24.77. LCMS R_T = 5.73 min; HRMS, calc'd for $C_{21}H_{18}F_4N_3O_4^+$ [M+H], 452.1228; found 452.1232.

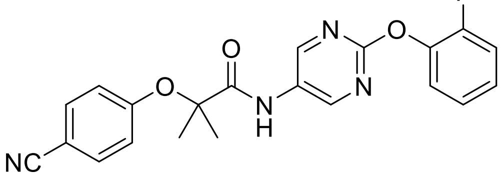

2-(4-Cyanophenoxy)-*N*-(2-(2-fluorophenoxy)pyrimidin-5-yl)-2-methylpropanamide (59). ^{1}H NMR (300 MHz, $CDCl_3$) *δ* 8.77 (s, 2H), 8.35 (s, 1H), 7.30 – 7.16 (m, 2H), 7.61 (d, *J* = 8.9 Hz, 4H), 7.08 – 7.01 (m, 2H), 1.66 (s, 6H) ; ^{13}C NMR (75 MHz, $CDCl_3$) δ 172.45, 161.30, 157.46, 156.22, 152.91, 151.51, 140.41, 140.24, 133.97, 126.88, 126.79, 124.79, 124.74, 123.64,

121.42, 118.39, 117.01, 116.77, 107.35, 82.73, 24.94. LCMS R_T = 5.37 min; HRMS, calc'd for $C_{21}H_{18}FN_4O_3^+$ [M+H], 393.1357; found 393.1353.

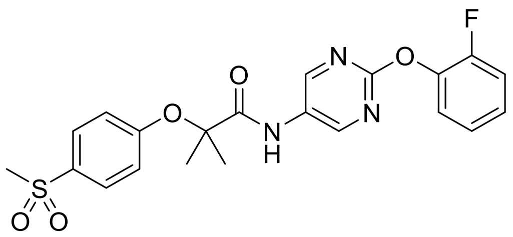

***N*-(2-(2-Fluorophenoxy)pyrimidin-5-yl)-2-methyl-2-(4-(methylsulfonyl)phenoxy)propanamide (60).** ^{1}H NMR (300 MHz, $CDCl_3$) δ 8.80 (s, 2H), 8.65 (s, 1H), 7.94 – 7.69 (m, 2H), 7.46 – 7.14 (m, 4H), 7.13 – 6.83 (m, 2H), 3.04 (s, 3H), 1.66 (s, 6H); ^{13}C NMR (75 MHz, $CDCl_3$) δ 172.59, 161.16, 158.46, 156.21, 152.91, 151.63, 140.40, 140.24, 135.08, 129.31, 128.84, 126.86, 126.76, 124.80, 124.75, 123.65, 121.09, 117.00, 116.76, 82.61, 44.67, 24.93. LCMS R_T = 4.68 min; HRMS, calc'd for $C_{21}H_{21}FN_3O_5S^+$ [M+H], 446.1180; found 446.1187.

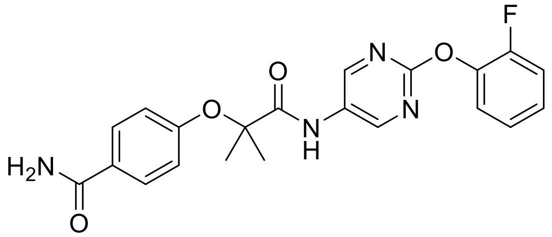

4-((1-((2-(2-Fluorophenoxy)pyrimidin-5-yl)amino)-2-methyl-1-oxopropan-2-yl)oxy)benzamide (61). ^{1}H NMR (300 MHz, $CDCl_3$) δ 8.81 (s, 2H), 7.86 – 7.56 (m, 2H), 7.23 (ddddd, J = 11.2, 8.3, 7.1, 5.4, 1.7 Hz, 5H), 7.06 – 6.90 (m, 2H), 1.64 (s, 6H); ^{13}C NMR (75 MHz, $CDCl_3$) δ 173.54, 169.48, 160.81, 157.31, 156.14, 152.84, 151.64, 140.14, 129.17, 127.98, 126.84, 126.75, 124.75, 124.70, 123.58, 119.98, 116.93, 116.69, 100.01, 81.69, 24.85. LCMS R_T = 4.29 min; HRMS, calc'd for $C_{21}H_{20}FN_4O_4^+$ [M+H], 411.1463; found 411.1464.

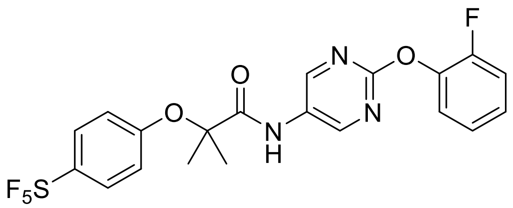

N-(2-(2-Fluorophenoxy)pyrimidin-5-yl)-2-methyl-2-(4-(pentafluoro-λ^6-sulfaneyl)phenoxy)propanamide (62). ^{1}H NMR (300 MHz, $CDCl_3$) δ 8.14 (s, 2H), 7.86 – 7.65 (m, 2H), 7.26 – 7.15 (m, 4H), 7.08 (d, J = 9.2 Hz, 2H), 1.70 (s, 6H); ^{13}C NMR (75 MHz, $CDCl_3$) δ 173.36, 156.34, 152.25, 149.33, 135.45, 127.73, 126.58, 126.48, 124.74, 123.75, 121.62, 116.97, 116.72, 100.01, 58.84, 26.05. LCMS R_T = 5.71 min; HRMS, calc'd for $C_{20}H_{18}F_6N_3O_3S^+$ [M+H], 494.0968; found 494.0844.

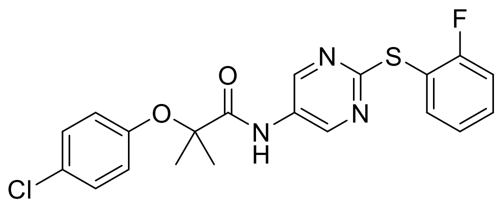

2-(4-Chlorophenoxy)-N-(2-((2-fluorophenyl)thio)pyrimidin-5-yl)-2-methylpropanamide (63). ^{1}H NMR (300 MHz, $CDCl_3$) δ 8.79 (s, 2H), 8.47 (s, 1H), 7.72 – 7.59 (m, 1H), 7.47 (dddd, J = 8.2, 7.5, 5.0, 1.7 Hz, 1H), 7.30 – 7.06 (m, 4H), 7.00 – 6.87 (m, 2H), 1.55 (s, 6H); ^{13}C NMR (75 MHz, $CDCl_3$) δ 173.10, 161.28, 151.87, 149.11, 136.86, 132.04, 131.93, 129.78, 129.59, 129.47, 129.37, 124.75, 124.70, 123.55, 118.22, 116.38, 116.08, 82.41, 24.76. LCMS R_T = 5.95 min; HRMS, calc'd for $C_{20}H_{18}ClFN_3O_2S^+$ [M+H], 418.0787; found 418.0794.

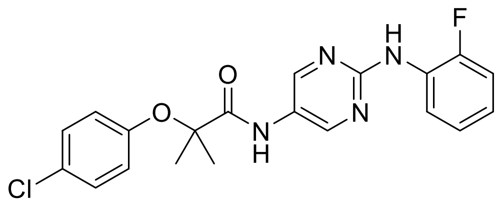

2-(4-Chlorophenoxy)-*N*-(2-((2-fluorophenyl)amino)pyrimidin-5-yl)-2-methylpropanamide (64). ^{1}H NMR (300 MHz, $CDCl_3$) δ 8.68 (s, 2H), 8.52 – 8.30 (m, 2H), 7.48 (d, *J* = 3.4 Hz, 1H), 7.38 – 7.22 (m, 2H), 7.16 – 7.02 (m, 2H), 7.01 – 6.77 (m, 3H), 1.57 (s, 6H); ^{13}C NMR (75 MHz, $CDCl_3$) δ 173.07, 156.87, 154.08, 152.15, 150.86, 150.66, 129.53, 129.49, 128.04, 127.92, 125.34, 124.36, 124.32, 123.39, 122.40, 122.30, 120.31, 120.30, 114.93, 114.68, 82.33, 24.85. LCMS R_T = 5.60 min; HRMS, calc'd for $C_{20}H_{19}ClFN_4O_2^+$ [M+H], 401.1175; found 401.118.

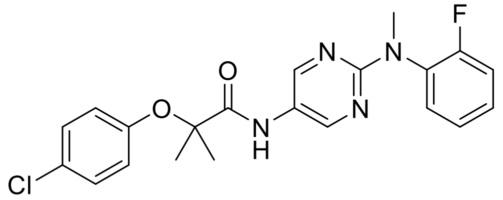

2-(4-Chlorophenoxy)-*N*-(2-((2-fluorophenyl)(methyl)amino)pyrimidin-5-yl)-2-methylpropanamide (65). ^{1}H NMR (300 MHz, $CDCl_3$) δ 8.54 (s, 2H), 8.27 (s, 1H), 7.37 – 7.28 (m, 1H), 7.27 – 7.08 (m, 5H), 7.02 – 6.83 (m, 2H), 3.49 (d, *J* = 0.6 Hz, 3H), 1.56 (s, 6H); ^{13}C NMR (75 MHz, $CDCl_3$) δ 172.97, 160.02, 159.22, 156.72, 152.27, 150.93, 133.04, 132.87, 129.49, 129.30, 129.18, 129.15, 128.09, 127.98, 124.75, 124.70, 123.58, 123.20, 116.80, 116.53, 82.26, 38.47, 38.45, 24.88. LCMS R_T = 5.66 min; HRMS, calc'd for $C_{21}H_{21}ClFN_4O_2^+$ [M+H], 415.1332; found 415.1337.

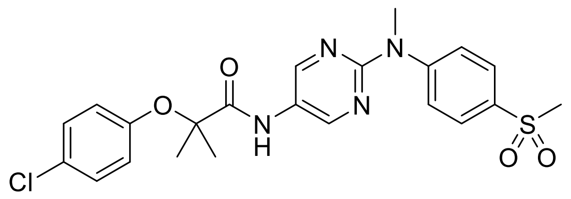

2-(4-Chlorophenoxy)-2-methyl-*N*-(2-(methyl(4-(methylsulfonyl)phenyl)amino)pyrimidin-5-yl)propanamide (67). ^{1}H NMR (300 MHz, $CDCl_3$) *δ* 8.65 (s, 2H), 8.43 (dd, *J* = 4.3, 1.2 Hz, 1H), 7.91 (dd, *J* = 8.8, 1.9 Hz, 2H), 7.56 (dd, *J* = 8.9, 1.2 Hz, 2H), 7.45 – 7.11 (m, 2H), 6.93 (dd, *J* = 9.0, 1.1 Hz, 2H), 3.63 (s, 3H), 3.07 (s, *J* , 3H), 1.57 (s, 6H); ^{13}C NMR (75 MHz, $CDCl_3$) δ 173.12, 158.34, 152.15, 150.45, 150.14, 135.31, 129.53, 129.46, 128.25, 125.09, 124.84, 123.34, 82.30, 44.71, 38.23, 24.86. LCMS R_T = 5.37 min; HRMS, calc'd for $C_{22}H_{24}ClFN_4O_4S^+$ [M+H], 475.1201; found 475.1203.

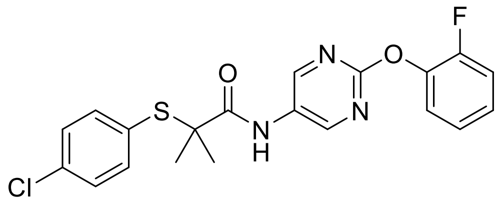

2-((4-Chlorophenyl)thio)-*N*-(2-(2-fluorophenoxy)pyrimidin-5-yl)-2-methylpropanamide (68). ^{1}H NMR (300 MHz, $CDCl_3$) *δ* 8.76 (s, 2H), 8.68 (s, 1H), 7.34 – 7.27 (m, 4H), 7.27 – 7.14 (m, 4H), 1.58 (s, 6H); ^{13}C NMR (75 MHz, $CDCl_3$) δ 172.89, 161.18, 156.26, 152.95, 151.42, 140.45, 140.28, 135.83, 135.74, 129.56, 129.32, 129.07, 126.85, 126.76, 124.79, 124.74, 123.68, 117.02, 116.78, 53.16, 26.47.. LCMS R_T = 5.61 min; HRMS, calc'd for $C_{20}H_{18}ClFN_3O_2S^+$ [M+H], 418.0787; found 418.0789.

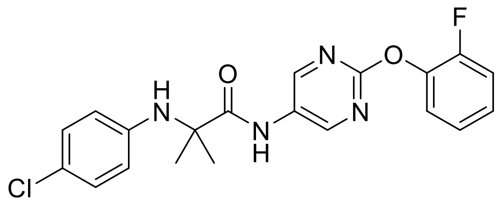

2-((4-Chlorophenyl)amino)-*N*-(2-(2-fluorophenoxy)pyrimidin-5-yl)-2-methylpropanamide (69). ^{1}H NMR (300 MHz, $CDCl_3$) δ 8.83 (s, 1H), 8.72 (s, 2H), 7.26 – 7.02 (m, 6H), 6.76 – 6.39 (m, 2H), 1.57 (s, 6H); ^{1} NMR (75 MHz, $CDCl_3$) δ 174.39, 161.11, 156.27, 152.97, 151.25, 142.61, 140.52, 140.36, 129.38, 129.07, 126.75, 126.66, 125.15, 124.73, 124.68, 123.68, 117.30, 116.98, 116.74, 58.86, 25.66. LCMS R_T = 5.36 min; HRMS, calc'd for $C_{20}H_{19}ClFN_4O_2^+$ [M+H], 401.1175; found 401.118.

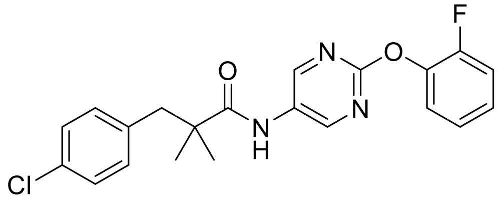

3-(4-Chlorophenyl)-*N*-(2-(2-fluorophenoxy)pyrimidin-5-yl)-2,2-dimethylpropanamide (73). ^{1}H NMR (300 MHz, $CDCl_3$) δ 8.60 (s, 2H), 7.30 – 7.15 (m, 6H), 7.05 (dd, J = 8.8, 2.3 Hz, 3H), 1.32 (s, 6H); ^{13}C NMR (75 MHz, $CDCl_3$) δ 175.58, 161.23, 156.24, 152.94, 152.30, 140.44, 140.28, 135.89, 132.82, 131.41, 128.66, 128.40, 126.84, 126.74, 124.77, 124.72, 123.69, 117.01, 116.77, 46.28, 44.27, 25.19. LCMS R_T = 5.57 min; HRMS, calc'd for $C_{21}H_{20}ClFN_3O_2^+$ [M+H], 400.1223; found 400.1227.

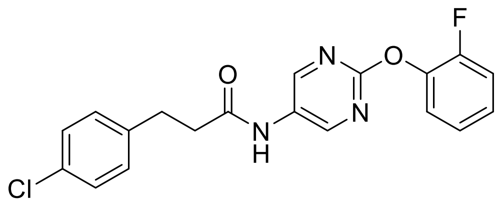

3-(4-Chlorophenyl)-*N*-(2-(2-fluorophenoxy)pyrimidin-5-yl)propanamide (74). ^{1}H NMR (300 MHz, $CDCl_3$) *δ* 8.67 (s, 2H), 8.19 (s, 1H), 7.25 – 7.14 (m, 5H), 7.23 – 7.06 (m, 3H), 2.98 (t, *J* = 7.5 Hz, 2H), 2.66 (t, *J* = 7.5 Hz, 2H); ^{13}C NMR (75 MHz, $CDCl_3$) δ 170.88, 160.78, 156.15, 152.85, 151.49, 140.27, 140.11, 138.77, 132.21, 129.72, 129.48, 128.74, 126.97, 126.88, 124.86, 124.81, 123.64, 117.03, 116.78, 38.35, 30.52. LCMS R_T = 5.12 min; HRMS, calc'd for $C_{19}H_{16}ClFN_3O_2^+$ [M+H], 374.0702; found 374.0695.

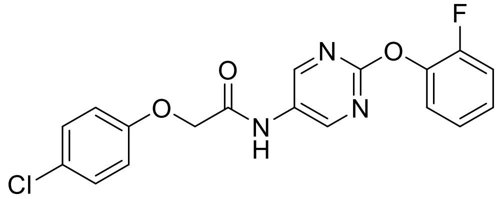

2-(4-Chlorophenoxy)-*N*-(2-(2-fluorophenoxy)pyrimidin-5-yl)acetamide (75). ^{1}H NMR (300 MHz, $CDCl_3$) *δ* 8.81 (s, 2H), 8.33 (s, 1H), 7.43 – 7.10 (m, 6H), 6.91 (d, *J* = 9.1 Hz, 2H), 4.62 (s, 2H); ^{13}C NMR (75 MHz, $CDCl_3$) δ 166.42, 161.43, 156.22, 155.30, 152.92, 151.87, 140.39, 140.23, 129.93, 128.13, 127.85, 126.90, 126.80, 124.79, 124.74, 123.66, 117.01, 116.77, 116.14, 67.65. LCMS R_T = 5.49 min; HRMS, calc'd for $C_{18}H_{13}ClFN_3O_3^+$ [M+H], 374.0702; found 374.0697.

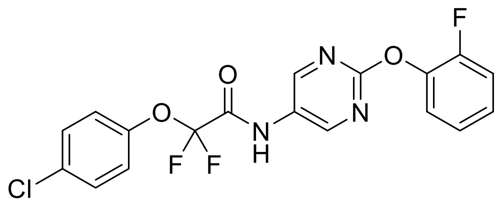

2-(4-Chlorophenoxy)-2,2-difluoro-*N*-(2-(2-fluorophenoxy)pyrimidin-5-yl)acetamide (79). ^{1}H NMR (300 MHz, $CDCl_3$) δ 8.85 (s, 2H), 8.46 (s, 1H), 7.41 – 7.32 (m, 2H), 7.30 – 7.11 (m, 6H); ^{13}C NMR (75 MHz, $CDCl_3$) δ 161.89, 158.11, 157.61, 157.11, 156.15, 152.84, 152.16, 147.37, 147.35, 147.32, 140.22, 140.06, 132.57, 129.93, 127.29, 127.09, 126.99, 124.85, 124.80, 123.59, 123.25, 117.77, 117.06, 116.82, 114.12. LCMS R_T = 5.50 min; HRMS, calc'd for $C_{18}H_{12}ClF_3N_3O_3^+$ [M+H], 410.0514; found 410.0516.

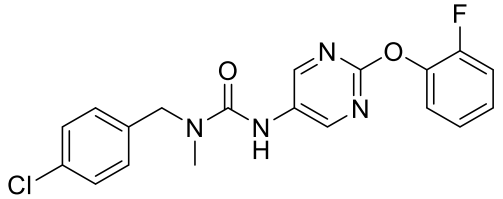

1-(4-Chlorobenzyl)-3-(2-(2-fluorophenoxy)pyrimidin-5-yl)-1-methylurea (82). ^{1}H NMR (300 MHz, $CDCl_3$) δ 8.56 (s, 2H), 7.37 – 7.28 (m, 2H), 7.26 – 7.12 (m, 6H), 6.68 (s, 1H), 4.53 (s, 2H), 2.99 (s, 3H); ^{13}C NMR (75 MHz, $CDCl_3$) δ 160.57, 156.24, 155.36, 152.94, 152.04, 140.53, 140.37, 135.53, 133.56, 130.30, 129.04, 128.81, 126.71, 126.61, 124.76, 124.71, 123.74, 116.96, 116.72, 51.78, 34.63. LCMS R_T = 5.07 min; HRMS, calc'd for $C_{19}H_{17}ClFN_4O_2^+$ [M+H], 387.1019; found 387.1025.

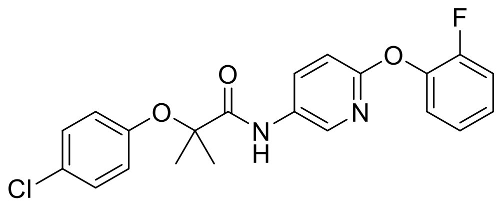

2-(4-Chlorophenoxy)-*N*-(6-(2-fluorophenoxy)pyridin-3-yl)-2-methylpropanamide (86). ^{1}H NMR (300 MHz, $CDCl_3$) δ 8.54 (s, 1H), 8.19 – 8.11 (m, 2H), 7.30 – 7.11 (m,6H), 7.00 – 6.86 (m, 3H), 1.55 (s, 6H); ^{13}C NMR (75 MHz, $CDCl_3$) δ 172.91, 159.69, 156.40, 153.10, 141.26, 141.10, 138.81, 132.18, 129.94, 129.52, 129.43, 126.11, 126.01, 124.69, 124.64, 123.77, 123.31, 117.02, 116.78, 110.80, 82.35, 24.86. LCMS R_T = 6.21 min; HRMS, calc'd for $C_{21}H_{19}ClFN_2O_3^+$ [M+H], 401.1063; found 401.1069.

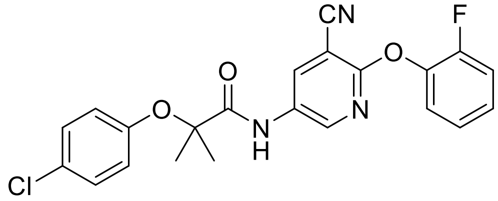

2-(4-Chlorophenoxy)-*N*-(5-cyano-6-(2-fluorophenoxy)pyridin-3-yl)-2-methylpropanamide (87). ^{1}H NMR (300 MHz, $CDCl_3$) δ 8.77 (s, 1H), 8.56 (d, *J* = 2.8 Hz, 1H), 8.31 (d, *J* = 2.8 Hz, 1H), 7.39 – 7.16 (m, 6H), 7.02 – 6.79 (m, 2H), 1.55 (s, 6H); ^{13}C NMR (75 MHz, $CDCl_3$) δ 173.28, 159.17, 156.07, 152.76, 151.93, 142.76, 139.96, 139.80, 134.81, 129.55, 127.19, 127.10, 123.81, 123.62, 117.03, 116.79, 114.24, 96.68, 82.34, 24.77. LCMS R_T = 6.09 min; HRMS, calc'd for $C_{22}H_{18}ClFN_3O_3^+$ [M+H], 426.1015; found 426.1022.

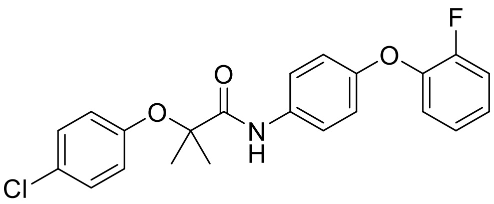

2-(4-Chlorophenoxy)-*N*-(4-(2-fluorophenoxy)phenyl)-2-methylpropanamide (88). ^{1}H NMR (300 MHz, $CDCl_3$) δ 8.48 (s, 1H), 7.63 – 7.48 (m, 2H), 7.30 – 7.21 (m, 2H), 7.21 – 7.02 (m, 4H), 7.00 – 6.88 (m, 4H), 1.57 (s, 6H); ^{13}C NMR (75 MHz, $CDCl_3$) δ 172.47, 155.81, 153.88, 152.49, 144.18, 144.03, 132.85, 129.48, 129.16, 124.71, 124.66, 124.62, 123.12, 121.47, 121.40, 121.38, 118.16, 117.23, 116.99, 82.33, 24.92. LCMS R_T = 6.10 min; HRMS, calc'd for $C_{22}H_{20}ClFNO_3^+$ [M+H], 400.1110; found 400.1117.

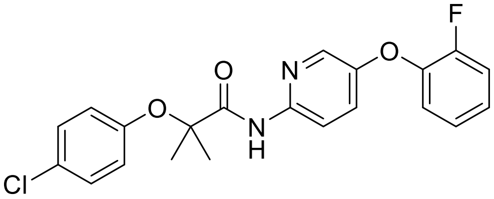

2-(4-Chlorophenoxy)-*N*-(5-(2-fluorophenoxy)pyridin-2-yl)-2-methylpropanamide (89). ^{1}H NMR (300 MHz, $CDCl_3$) δ 9.12 (s, 1H), 8.26 (dd, *J* = 9.0, 0.7 Hz, 1H), 8.08 (dd, *J* = 3.0, 0.7 Hz, 1H), 7.35 (dd, *J* = 9.1, 2.9 Hz, 1H), 7.27 – 7.23 (m, 4H), 7.21 – 7.00 (m, 2H), 6.96 – 6.88 (m, 2H), 1.58 (s, 6H); 13C NMR (75 MHz, CDCl3) δ 172.85, 155.66, 152.42, 150.71, 146.46, 143.61, 143.46, 137.95, 129.43, 129.15, 129.12, 127.14, 125.40, 125.31, 124.92, 124.87, 123.13, 121.26, 117.48, 117.24, 114.34, 82.02, 24.84. LCMS R_T = 6.17 min; HRMS, calc'd for $C_{21}H_{19}ClFN_2O_3^+$ [M+H], 401.1063; found 401.1059.

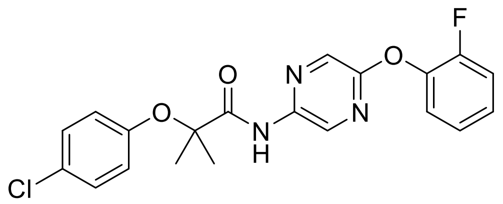

2-(4-Chlorophenoxy)-*N*-(5-(2-fluorophenoxy)pyrazin-2-yl)-2-methylpropanamide (90).

^{1}H NMR (300 MHz, $CDCl_3$) δ 9.09 (d, *J* = 1.5 Hz, 1H), 9.05 (s, 1H), 8.17 (d, *J* = 1.5 Hz, 1H), 7.29-7.27 (m, 1H), 7.26 – 7.15 (m, 5H), 6.95 – 6.88 (m, 2H), 1.57 (s, 6H); ^{13}C NMR (75 MHz, $CDCl_3$) δ 172.66, 152.25, 143.15, 132.22, 131.72, 129.49, 129.34, 126.67, 126.56, 124.83, 124.77, 123.63, 123.21, 117.15, 116.91, 82.06, 76.61, 24.81. LCMS R_T = 6.04 min; HRMS, calc'd for $C_{20}H_{18}ClFN_3O_3^+$ [M+H], 402.1015; found 402.1019.
